# Novel Inhibitors Induce Large Conformational Changes of GAB1 Pleckstrin Homology Domain and Kill Breast Cancer Cells

**DOI:** 10.1371/journal.pcbi.1004021

**Published:** 2015-01-08

**Authors:** Lu Chen, Lei Du-Cuny, Sylvestor Moses, Sabrina Dumas, Zuohe Song, Abdol Hossein Rezaeian, Hui-Kuan Lin, Emmanuelle J. Meuillet, Shuxing Zhang

**Affiliations:** 1 Department of Experimental Therapeutics, The University of Texas M. D. Anderson Cancer Center, Houston, Texas, United States of America; 2 Departments of Nutritional Sciences and Molecular and Cellular Biology, the University of Arizona Cancer Center, Tucson, Arizona, United States of America; 3 Department of Molecular and Cellular Oncology, The University of Texas M.D. Anderson Cancer Center, Houston, Texas, United States of America; University of California San Diego, United States of America

## Abstract

The Grb2-associated binding protein 1 (GAB1) integrates signals from different signaling pathways and is over-expressed in many cancers, therefore representing a new therapeutic target. In the present study, we aim to target the pleckstrin homology (PH) domain of GAB1 for cancer treatment. Using homology models we derived, high-throughput virtual screening of five million compounds resulted in five hits which exhibited strong binding affinities to GAB1 PH domain. Our prediction of ligand binding affinities is also in agreement with the experimental *K*_D_ values. Furthermore, molecular dynamics studies showed that GAB1 PH domain underwent large conformational changes upon ligand binding. Moreover, these hits inhibited the phosphorylation of GAB1 and demonstrated potent, tumor-specific cytotoxicity against MDA-MB-231 and T47D breast cancer cell lines. This effort represents the discovery of first-in-class GAB1 PH domain inhibitors with potential for targeted breast cancer therapy and provides novel insights into structure-based approaches to targeting this protein.

## Author Summary

In this paper, we described the identification and evaluation of a set of first-in-class potent inhibitors targeting a new cancer target, Grb2-associated binder-1 (GAB1), which integrates signals from different signaling pathways, and is frequently over-expressed in cancer cells. To achieve our goals, we have employed intensive computational modeling to understand the structure of the GAB1 pleckstrin homology (PH) domain and screened five million compounds. Upon biological evaluation, we found that several inhibitors that induced large conformational changes of the target structure exhibited strong selective binding to GAB1 PH domain. Particularly, these inhibitors demonstrated potent and tumor-specific cytotoxicity in breast cancer cells. This represents a groundbreaking discovery in targeting GAB1 signaling which may be used for cancer therapy, especially for triple negative breast cancer patients.

## Introduction

Overexpression of Grb2-associated binding protein 1 (GAB1) has been observed in several human cancers, such as breast and lung cancers [1]–[4]. This protein is a substrate of several growth factors and interleukin receptors, and it is involved in the integration of different signal transductions [1]–[4]. Particularly, GAB1 mediates the activation of mitogen-activated protein kinase (MAPK) and phosphoinositide 3-kinase (PI-3K) cascades [5], [6]. It belongs to a family of scaffolding proteins closely related to the insulin receptor substrates (e.g., IRS1) [2]. It contains an N-terminal pleckstrin homology (PH) domain binding to phosphatidylinositol-(3,4,5)-triphosphate (PtdIns(3,4,5)P_3_), tyrosine phosphorylation sites for the Src homology 2 (SH2) binding and a proline-rich domain interacting with Src homology 3 (SH3) domain [6]. PH domains can be subdivided into four groups based on their selective binding to phosphoinositides [7], and GAB1 PH domain belongs to Group 1 which exhibits the strongest binding to PtdIns(3,4,5)P_3_, but weak affinity and specificity to PtdIns(3,4)P_2_ or PtdIns(4,5)P_2_ [4], [8]. Additionally, the phosphorylation of GAB1 on Y627 depends on the intracellular translocation from cytosol to membrane by binding to PtdIns(3,4,5)P_3_ via its PH domain [9]. Therefore, inhibition of GAB1 PH domain functions may prevent the recruitment of GAB1 to the membrane and suppress cancer cell (e.g., breast cancer) proliferation and metastasis [10]. Herein, we attempt to identify novel small molecule inhibitors selectively targeting the PH domain of GAB1 and suggest that these small molecules exhibit high therapeutic potency for cancer treatment.

**Figure 1:**
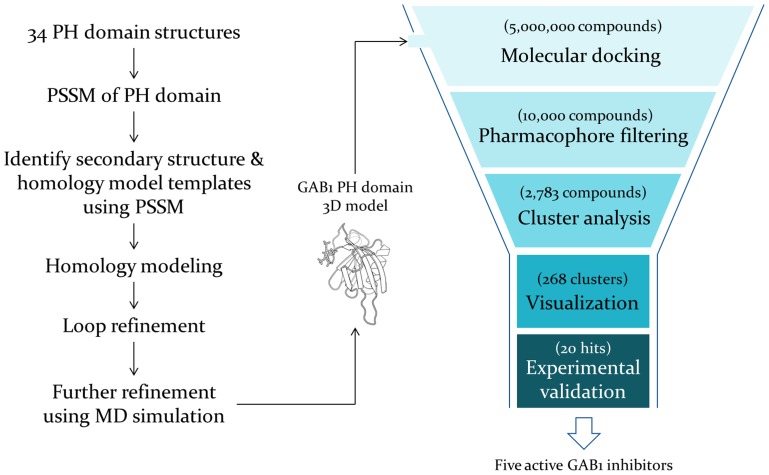
Structure-based drug discovery workflow. All of the available PH domain 3D structures in the PDB were used to build our PSSM scoring functions which were employed to construct the homology model of GAB1 PH domain. The derived structure was used to perform high-throughput virtual screening of five million drug/lead-like compounds. Through a funnel-like process 20 hits were selected for experimental testing and five were confirmed as consistently active in all of the assays.

Unfortunately, no three-dimensional (3D) structure is available to date for GAB1 PH domain or any PH domain in complex with drug-like small molecules. Challenges remain for accurate structural prediction due to its low sequence identity (<30%) to other PH domains with known structures [11]. However, the core β-sandwich fold among PH domains is conserved [11], making it possible to construct a reliable homology model structure of GAB1 PH domain. Here, based on the position-site specific matrixes (PSSM) obtained from all non-redundant PH domain structures, we performed fold recognition and homology modeling, followed by intensive structural refinement. The resulted model was then applied to high-throughput virtual screening of our unique collection of over five million drug and lead-like compounds with our in-house drug discovery workflow (**Fig. 1**) [12]. Upon biological evaluation, five out of the initially tested 20 hits exhibited positive activities to form direct binding to GAB1 PH domain, inhibit GAB1 Y627 phosphorylation and suppress breast cancer cell proliferation with low micromolar IC_50_. As is known, triple negative breast cancers are more aggressive with poor prognosis and difficult to treat clinically [13], but our inhibitors showed high potency against these malicious cells. Therefore, this study validates the effectiveness of our *in silico* platform for drug discovery, and demonstrates that targeting the PH domain of GAB1 provides a promising and novel therapeutic strategy for cancer treatment.

## Results

### Fold recognition and sequence alignment

PH domains are unique due to their conserved secondary structures and 3D folds, all with seven β-sheets and a C-terminal helix. However, the pairwise sequence identities among different PH domains are usually below 30%, and the loop regions are hypervariable in length and amino acid sequence [11]. Herein, we collected all available 34 non-redundant crystal structures of PH domains from Protein Data Bank (PDB) [14] and performed secondary structure-based sequence alignment using STRAP [15]. From the sequence alignment, we generated PSSMs for β_1_, β_2_, β_3_, β_6_, β_7_ and α_1_ (presented as sequence logos in **S1 Fig.**) to guide secondary structure prediction of new PH domain (e.g., GAB1). As no reliable PSSMs for β_4_ and β_5_ were generated due to low sequence similarity, we used PSIPRED server [16] to predict these two β-sheets.

**Figure 2:**
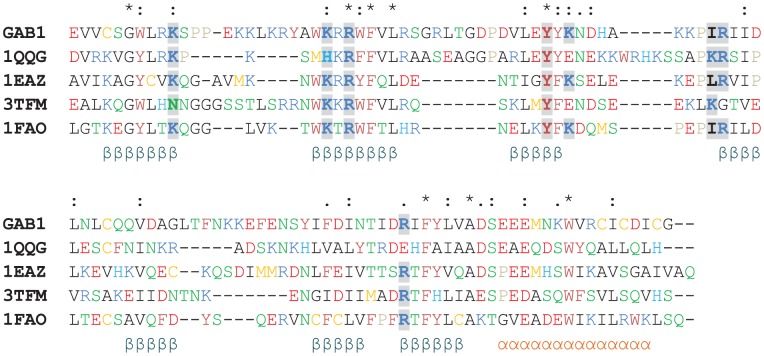
Sequence alignment of the PH domains. IRS1 (PDB ID: 1QQG), TAPP1 (PDB ID: 1EAZ), Myosin X (PDB ID: 3TFM) and DAPP1 (PDB ID: 1FAO). The secondary structure of the generated GAB1 homology model and the crystal structures of the templates are illustrated, using α for α-helix, β for β-sheet. Except for the highly variable β_1,2_ loop regions, the active site residues directly interacting with PtdIns(3,4,5)P_3_ are highlighted with shadows.

**S1 Fig.** shows the sequence logos derived from the collected 34 PH domains, in which the size of residue indicates the relative frequency of that residue at the corresponding position. As expected, we found that most conserved residues are in the hydrophobic cores of PH domains. The residues responsible for phosphoinositide binding are generally located at β_1_[7], β_2_[2], β_2_[5], β_3_[4], β_3_[+1] and β_7_ [1] (the number in the brackets indicates the residue position at the secondary structure element). Predominantly, they are basic residues such as lysine and arginine. We combined these observations with PSSM and PSIPRED to predict the secondary structure of GAB1 PH domain, and found the predicted structure preserves a typical β-sandwich fold where C8-K14, W26-L33, V44-Y48, R58-D61, Q66-G71, I84-N88, and R92-V97 form the respective seven β-sheets, while E101-I114 forms the C-terminal α-helix (**Fig. 2**). However, the GAB1 PH domain is unique with: 1) a long β_1,2_ loop landmarked by the conserved K14 and W26, similar to myosin X (PDB ID: 3TFM [17]); 2) a long β_2,3_ loop, similar to IRS1 (PDB ID: 1QQG [18]); 3) a long β_5,6_ loop, similar to TAPP1 (PDB ID: 1EAZ [19]); 4) the highest sequence identity of active-site residues (except for β_1,2_ loop region) to DAPP1 (PDB ID: 1FAO [20]) (shadowed residues in **Fig. 2**). Therefore, we have chosen the above four proteins as the templates for the following-up homology modeling studies.

### Homology modeling and structural optimization with molecular dynamics

We constructed 1,000 homology models of GAB1 PH domain in complex with inositol-tetrakisphosphate (IP4) based on the X-ray crystal structures of four aforementioned templates. After loop refinement and molecular dynamics (MD) simulation, we selected one reliable model in which IP4 binds stably to GAB1 PH domain with a minor fluctuation of phosphates (RMSF<1.1 Å), shown in **S2 Fig**. The simulation of this model reached the equilibrium after 5 ns, as judged by the RMSD of all of the backbone atoms (C, CA and N) (**S2 Fig.**). Large fluctuations of the Cα atoms were only observed in the β_1,2_, β_2,3_ and β_5,6_ loops (**S2 Fig.**). The quality of the lowest-energy model was assessed by QMEAN [21], ProSA [22] and PROCHECK [23]. The Ramachandran plot showed reasonable backbone dihedral angles: 92.2% of the residues were in the most favored regions, and eight residues in the additional or generously allowed regions. Both the ProSA Z-score (−4.04) and QMEAN Z-score (−0.13) of final model were within the range as typically seen for the native proteins of the similar size (**S3 Fig.**). In addition, the DOPE per-residue profile demonstrated a significant decrease in the DOPE scores at the β_2,3_ loop, β_4,5_ loop, β_5_, β_5,6_ loop and β_6_ for the refined structure compared with the initial homology model (**S4 Fig.**, and homology model coordinate file is available at http://imdlab.org/supporting/PLOSCompBio).

**Figure 3:**
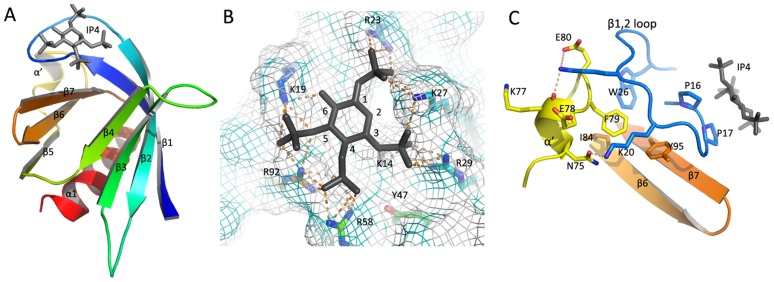
The 3D model of GAB1 PH domain. **A.** The overall structure of IP4-bound GAB1 PH domain. The protein secondary structure is shown in ribbons and IP4 is shown in sticks. **B.** A close view of the IP4 binding site. The critical residues for IP4 binding are labeled, and IP4 is in black stick with position labeled. The hydrogen bonds are illustrated with dashes. **C.** Interactions between α′-helix and β_5,6_ loop. The residues mediating the interactions are highlighted with sticks and hydrogen bonds are illustrated with dashes.

As illustrated by **Fig. 3A**, the 3D model of GAB1 PH domain maintained the conserved β-sandwich folding. Similar to other Group 1 PH domains (e.g., Grp1 [20] and Btk [24]), the phosphoinositide-binding site of GAB1 was surrounded by the β_1,2_, β_3,4_ and β_6,7_ loops. The 2-hydroxyl group of IP4 oriented towards the β_1,2_ loop, and the 3,4,5-phosphates intensively interacted with the aforementioned basic residues in the β_1_, β_2_, β_4_ and β_7_. Particularly, K19 and R23 in the β_1,2_ loop formed hydrogen bonds with 5-P and 1-P, respectively (**Fig. 3B**). This explains why GAB1 PH domain specifically binds to PtdIns(3,4,5)P_3_ but not PtdIns(3,4)P_2_ or PtdIns(4,5)P_2_. Strikingly, the sequence motif NKKEFE in the β_5,6_ loop folded into an additional α-helix, as we termed α′. This additional α-helix also occurs in phospholipase Cδ PH domain (PDB ID: 1MAI [25]), and it interacts with W26, F79 and Y95 in the β_1,2_ loop via hydrogen bonding networks and hydrophobic interactions (**Fig. 3C**). This α′-helix was likely to stabilize the IP4-bound conformation of β_1,2_ loop, as W26A or W26C mutation impairs the PtdIns(3,4,5)P_3_ binding [8]. Furthermore, the motif SPP in the β_1,2_ loop formed intensive vdW interactions with the β_7_ and inositol scaffold (**Fig. 3C**). Finally, GAB1 PH domain had an extra hydrophobic region (later defined as *Region II*) due to the smaller side chains of those hydrophobic residues around β_6,7_ loop compared to IRS1. All these specific structural features intrinsically offered possibility of designing selective inhibitors against GAB1 over other PH domains, as further discussed in the ligand-induced conformational changes section.

### *In silico* hit identification

To identify novel inhibitors of GAB1 PH domain, we performed structure-based virtual screening using our MD-refined structural model. Additionally, a protein-based pharmacophore filter was derived using GRID method to select those high-throughput virtual screening hits of which the docked poses matched the pharmacophores [26], [27]. Residues K14, R23, K27, R29, R58 and R92 were identified as the residues that favorably interact with hydrogen bond acceptors, whereas Y47, F94 and I60 were specified as the preferential areas for hydrophobic moieties (**S5 Fig.**). The residues responsible for PtdIns(3,4,5)P_3_ binding were predicted to be K14, K27, R29, Y47, K49, R58 and R92, consistent to the mutagenesis studies [6], [8]. These critical residues were employed to define the protein pharmacophores to select docking poses of those 10,000 top ranked hits (only based on docking scores ranging 43.47–101.39) from the virtual screening of over five million compounds of our in-house collection. The resulted 2,783 hits were subjected to cluster analysis based on their chemical diversity (Tanimoto coefficient <0.65), and we obtained 268 clusters and selected the best-scored hits from each cluster (**Fig. 1**). Upon visualizing their molecular interactions with the GAB1 PH domain, we chose 20 hits as listed in **Table 1** and **S1 Table**.

**Table 1:** Biochemical and biological activities of hits.

	K_D_ or K_i_ (µM)			% pGAB1 inhibition	IC_50_ (µM)	
ID	GAB1	IRS1	AKT1		MDA-MB-231	T47D
GAB-001	9.38±1.60*	NB	ND	55	40.3±1.3	23.7±2.7
GAB-002	2.1±0.1	16.4±4.3	ND	32	142.7±11.6	137.6±6.9
GAB-003	2.7±0.8	3.3±1.5	ND	14	85.2±7.2	13.9±0.6
GAB-004	2.2±1.1	NB	ND	81	66.6±4.6	19.4±2.9
GAB-005	NB	NB	ND	6	IA	IA
GAB-006	NB	NB	ND	16	ND	100.8±1.8
GAB-007	42.3±8.9	1.6±0.2	ND	53	4.6±0.7	20.1±4.3
GAB-008	NB	16.2±1.7	ND	8	IA	43.2±3.2
GAB-009	NB	NB	ND	38	162.9±6.0	97.6±6.1
GAB-010	NB	0.12±0.02	ND	92	66.0±4.4	23.8±4.2
GAB-011	NB	NB	ND	24	IA	IA
GAB-012	NB	NB	4.58±1.72	43	IA	119.2±3.6
GAB-013	NB	NB	6.27±1.16	27	IA	IA
GAB-014	NB	NB	NB	42	69.9±0.1**	177.5±3.9
GAB-015	13.1±1.5*	0.05	5.0±0.4*	77	71.7±1.6	125.4±5.4
GAB-016	0.9±0.1	0.15	4.3±0.1*	96	79.0±1.1	45.9±1.6
GAB-017	0.68±0.03	0.05	18.9±1.2*	82	40.6±0.8	41.4±2.7
GAB-018	36.6±5.0*	NB	6.0±1.0*	71	85.3±1.5	185.4±2.5
GAB-019	ND	ND	ND	91	ND	39.4±6.2
GAB-020	1.33±4.5	ND	2.4±0.6*	87	ND	68.2±5.5
DPIEL	2.8	ND	5.04±0.48	72	ND	30.6±4.3
IP4	ND	ND	3.08±0.49	ND	ND	ND

*Ki measurement.

**MDA-MB-468 cell line used.

ND: Not Determined. NB: No Binding. IA: Inactive.

### Biological evaluation of identified hits

**Figure 4:**
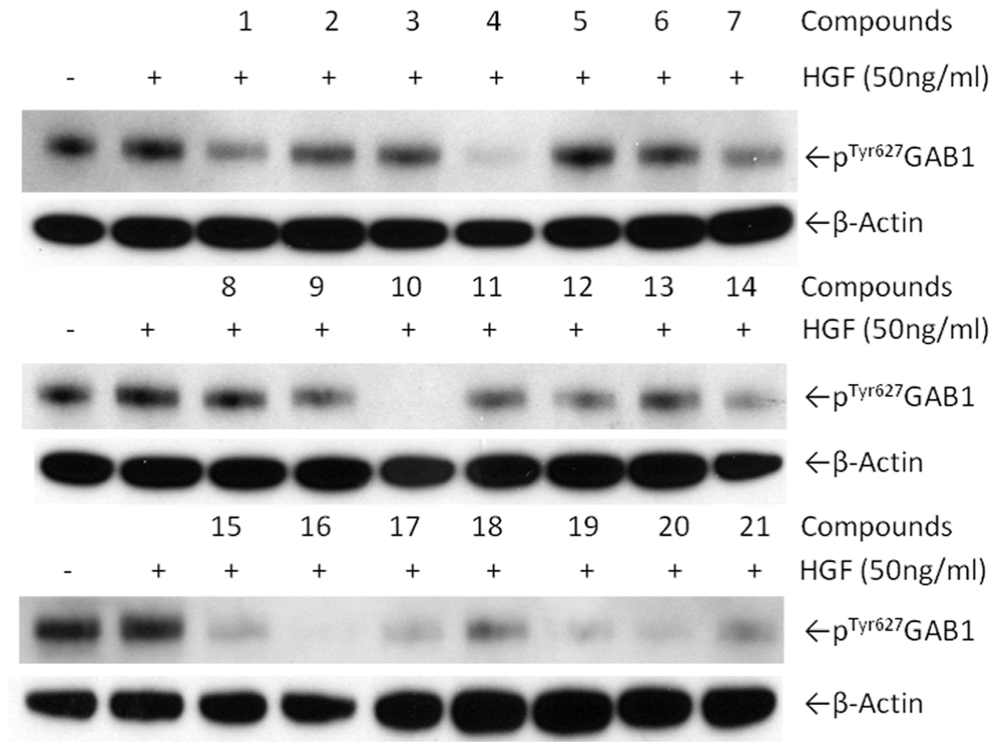
Inhibition of Y627 phosphorylation in GAB1. *No.* 1-20 are GAB-001 to GAB-020, and *No.* 21 is DPIEL.

**Figure 5:**
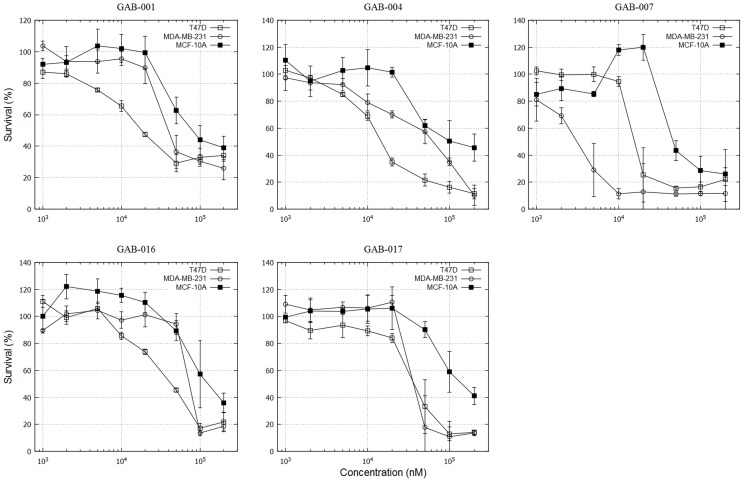
Cell proliferation assay for T47D and MDA-MB-231 breast cancer cell line and MCF-10A breast epithelial cell line.

To validate our *in silico* identified hits, we performed three types of experimental assays to evaluate their bioactivities: direct binding to GAB1 PH domain, inhibition of Y627 phosphorylation of GAB1, and cytotoxicity IC_50_ in triple negative MDA-MB-231 and T47D human breast cancer cells. Our experiments revealed that 10 out of 20 hits demonstrated submicromolar to micromolar binding affinity (<50 µM) to GAB1 PH domain measured by surface plasmon resonance (SPR). Among them, GAB-001, GAB-004, GAB-007, GAB-016 and GAB-017 demonstrated promising bioactivity in the subsequent *in vitro* assays (**Table 1**, **Fig. 4** and **Fig. 5**).

GAB-001 exhibited selective binding to GAB1 (*K*_i_ = 9.4±1.6 µM) (**S6 Fig.**), but not to IRS1 PH domain. In addition, it inhibited Y627 phosphorylation and killed breast cancer cells at IC_50_ = 23.7±2.7 µM (for T47D). GAB-004 achieved similar binding selectivity as GAB-001, but had a stronger inhibition of pY627 (81%) and a lower IC_50_ (19.4±2.9 µM). Interestingly GAB-007 demonstrated weak binding (*K*_D_ = 42.3±8.9 µM) and mild pY627 inhibition (53%), but it showed high cytotoxicity in both MDA-MB-231 (IC_50_ = 4.6±0.7 µM) and T47D (IC_50_ = 20.1±4.3 µM) cell lines (**Fig. 5**), probably due to other off-target mechanisms. GAB-016 and GAB-017 are N-(1,3,4-thiadiazol-2-yl)benzenesulfonamide derivatives which were previously synthesized through our search for AKT PH domain inhibitors [28]. They demonstrated nanomolar binding affinity for GAB1 PH domain (**S6 Fig.**), and were 5-fold and 28-fold more selective to the GAB1 than AKT, respectively. Consistent to their high binding affinities, GAB-016 and GAB-017 also inhibited over 80% of Y627 phosphorylation.

### GAB1 targeted tumor-specific cytotoxicity

All the aforementioned active inhibitors showed potent cytotoxicity to cancer cell lines (T47D and MDA-MB-231). More excitingly the cytotoxicity is specific to cancer cells as the inhibitors exhibit little inhibition in the non-cancer MCF-10A breast cell line (**Fig. 5**). Expectedly, as GAB1 and IRS1 pathways are intertwined [29], some inhibitors could suppress IRS1 phosphorylation (**S7 Fig.**). In addition, some compounds that selectively bind AKT PH domain (e.g., GAB-012, GAB-013 and GAB-018) did not effectively kill MDA-MB-231 or T47D breast cancer cell lines at 50 µM (**Table 1**).

### MD simulation of protein-ligand complexes and binding free energy calculation

To further investigate the structural mechanisms of our inhibitors to interact with the GAB1 PH domain, we performed MD simulations of the protein-inhibitor complexes (listed in **Table 2**). As expected, the active compounds (GAB-001, GAB-004, GAB-007, GAB-016 and GAB-017) demonstrated stable bindings to GAB1 PH domain in three independent simulations (RMSD<2.5 Å), whereas GAB-002 and GAB-003 dissociated with the protein after around 25 ns (**S8 Fig.**). In addition, MD simulations showed that GAB-007, GAB-010 and GAB-016 could form stable binding to IRS1 PH domain (**S8 Fig.**), consistent to the SPR results in **Table 1**.

**Table 2:** Computation of the absolute binding free energy using PMF-based routine.

	GAB1				
Component	GAB-001	GAB-004	GAB-007	GAB-016	GAB-017
Δ*Gc,site* (kcal/mol)	1.14	0.98	0.92	1.42	1.31
Δ*Gc,bulk* (kcal/mol)	1.27	1.00	1.09	1.52	1.65
Δ*Go,bulk* (kcal/mol)	1.04	0.9	0.85	0.98	0.67
S* (Å^2^)	6.27×10^3^	8.74×10^3^	9.94×10^3^	7.00×10^3^	7.91×10^3^
I* (Å)	8.52×10^5^	3.76×10^5^	1.53×10^4^	1.32×10^7^	3.43×10^6^
Δ*G_bind_* (kcal/mol)	−7.76	−7.72	−5.79	−9.55	−8.89
Exp. Δ*G_bind_* (kcal/mol)	−6.91	−7.78	−6.01	−8.31	−8.47

	IRS1		
Component	GAB-007	GAB-010	GAB-016
Δ*Gc,site* (kcal/mol)	1.02	0.84	1.26
Δ*Gc,bulk* (kcal/mol)	1.08	1.07	1.46
Δ*Go,bulk* (kcal/mol)	1.14	0.85	0.94
S* (Å^2^)	8.63×10^3^	5.75×10^3^	6.81×10^3^
I* (Å)	1.04×10^6^	1.18×10^7^	4.90×10^7^
Δ*G_bind_* (kcal/mol)	−8.05	−9.36	−10.26
Exp. Δ*G_bind_* (kcal/mol)	−7.93	−9.51	−9.38

**Figure 6:**
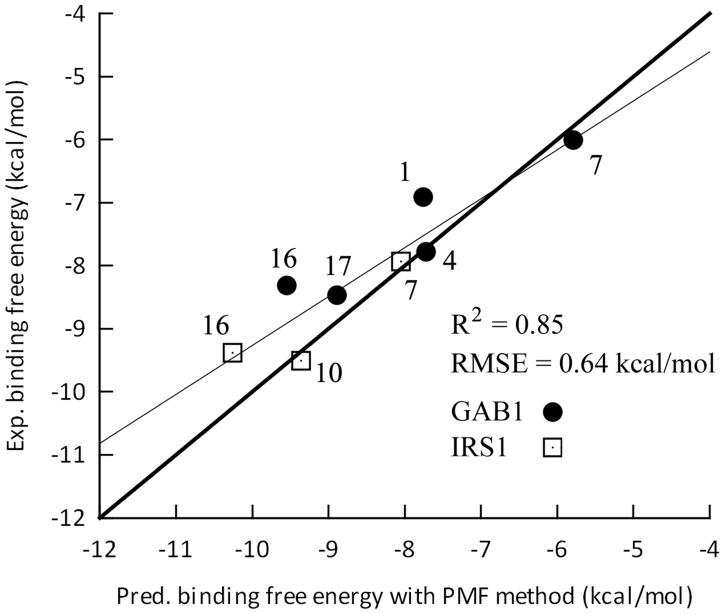
The correlation between the predicted binding free energies (PMF method) and the experimental ones. The grey line indicates the calculated correlation between predicted and experimental binding free energies, and the black line indicates the ideal correlation. The number beside each point is the corresponding inhibitor ID.

To add another layer of validation of the binding modes predicted by MD simulations, we calculated the absolute binding free energies of our inhibitors to GAB1/IRS1 PH domain using an in-house potential of mean force (PMF) method, which aims to circumvent the insufficient sampling issue by introducing hypothetical intermediate states representing the association pathway of ligand from the unbound “bulk” regions to the ligand-binding “site” (**S9 Fig.**). The principle of this approach has been described elsewhere [30], [31]. Here, we implemented this method using ff99SB force field [32]. Briefly, the umbrella sampling and weighted histogram analysis were used as the primary tools to derive two sets of PMF: ligand conformational PMF *w*(ξ) and protein-ligand separation PMF *w*(r). The details of mathematical calculations were available in **S1 Method**, and the *w*(ξ) and *w*(r) plots for eight protein-ligand complexes were available in **S10 Fig.** and **S11 Fig.** As indicated by **Fig. 6** and **Table 2**, the predicted absolute binding free energies via PMF method were in a good agreement with the experimental values (RMSE = 0.64 kcal/mol, R^2^ = 0.85). One may notice that these predictions encompassed two different PH domain targets (GAB1 and IRS1) and a variety of ligand chemotypes. The good correlation between experimental binding free energies and predicted free energies implied that the predicted inhibitor binding modes by MD simulations were accurate.

### Ligand-induced conformational changes of PH domain

We have generated eight reliable PH domain-inhibitor complex models from MD simulations (listed in **Table 2**) which have been validated by the PMF absolute binding free energy calculations as described in the previous section. When comparing the bound and unbound protein structures, we observed for the first time the ligand-induced conformational changes in three regions around the phoshpoinositide-binding pocket (termed as *Region I*, *Region II* and *Region III*) for both GAB1 and IRS1 PH domains.

**Figure 7:**
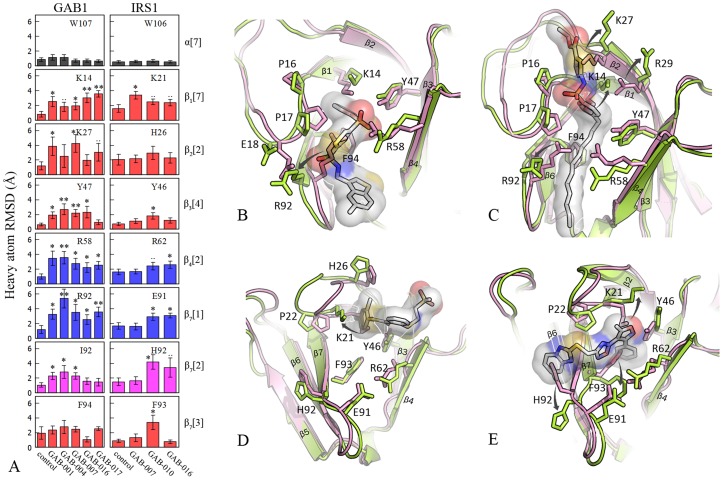
Inhibitor-induced conformational changes for PH domain structures. **A.** Heavy atom RMSDs for the residues with conformational changes. RMSD of each residue is calculated from the snapshots taken from three independent MD simulations and compared with the unbound control structure. W107_GAB_1 and W106_IRS_1 (in black) are used as the controls for no conformational change. Red: *Region I* residues; Blue: *Region II* residues; Magenta: *Region III* residues. “..” for p<0.1; “*” for p<0.05; “**” for p<0.01. **B–E**. The critical residues in the three regions are shown in sticks. Pink: the protein in unbound state; Green: the protein in inhibitor-bound state. The inhibitors are depicted with gray sticks and surfaces. The arrows illustrate large conformational changes. **B.** GAB1 PH domain in complex with GAB-001; **C.** GAB1 PH domain in complex with GAB-017; **D.** IRS1 PH domain in complex with GAB-007; **E.** IRS1 PH domain in complex with GAB-010.

The *Region I* is comprised of the conserved K14_GAB1_/K21_IRS1_ (β_1_[7]), K27_GAB1_/K21_IRS1_ (β_2_[2]), Y47_GAB1_/Y46_IRS1_ (β_3_[4]) and F94_GAB1_/F93_IRS1_ (β_7_[3]) (**Fig. 7A**–**E**). The MD simulations showed significant conformational changes in *Region I* (RMSD>2 Å) for both GAB1 and IRS1, as illustrated by the RMSD analysis (red plots in **Fig. 7A**). The side chain rearrangement of these residues, especially K14_GAB1_/K21_IRS1_ and Y47_GAB1_/Y46_IRS1_, created a pocket which favorably binds an aromatic moiety connecting with a H-bond acceptor group (**Movie S1, S2, S3** available at http://imdlab.org/supporting/PLOSCompBio). This moiety could form cation-π and hydrophobic interactions with the surrounding K14_GAB1_/K21_IRS1_ and F94_GAB1_/F93_IRS1_, respectively (**Fig. 7B–E**). All inhibitors we identified in this study contain such a pharmacophore (phenylthiazole in GAB-004, phenylisoxazole in GAB-010, S-phenyl carbothioate in GAB-007, benzenesulfone in GAB-001, GAB-016 and GAB-017) (**S12 Fig.**). We had mentioned that β_1_[7], β_2_[2] and β_3_[4] were the PIP3-binding residues, thus *Region I* conformational changes were attributable to the activities of the inhibitors. Generally, the conformational changes of *Region I* residues in GAB1 were more substantial than IRS1, except F94_GAB1_ (**Fig. 7A**). In comparison, GAB-010 could induce an alternative conformation of F93_IRS1_ (**Fig. 7E**), which also occurred in ArhGAP9 crystal structure (PDB ID: 2P0D [33]). The function of conformational change of F93_IRS1_ is likely to further open the pocket to accommodate larger moiety such as phenylisoxazole (GAB-010), as other IRS1 *Region I* residues were less flexible.

The *Region II* is formed by β_4_, β_6,7_ loop and the first several amino acids of β_7_ (**Fig. 7A–E**). The key residues are R58_GAB1_/R62_IRS1_ (β_4_[2]) and R92_GAB1_/E91_IRS1_ (β_7_[1]). Compared with *Region I* residues, more significant conformational changes were observed in the *Region II* residues in the GAB1 PH domain (RMSD>2.5 Å) (blue plots in **Fig. 7A**). These conformational changes created a new pocket which binds aliphatic (GAB-016 and GAB-017) and aromatic moieties (e.g., chlorobenzothiophene in GAB-001 and furan in GAB-004). Remarkably, we found that the bulky aromatic moieties (GAB-001 and GAB-004) generally induced more movement of GAB1 *Region II* residues than the aliphatic moieties (GAB-016 and GAB-017) (blue plots in **Fig. 7A**). We also observed that significant conformational changes only occurred in GAB1, but not in IRS1 PH domain (blue plots in **Fig. 7A**), probably because the electrostatic attraction between R62_IRS1_ and E91_IRS1_ significantly restrained the fluctuation of these two residues (**Movie S1** available at http://imdlab.org/supporting/PLOSCompBio), while the electrostatic repelling between R58_GAB1_ and R92_GAB1_ made these two residues more flexible (**Movie S3**). These findings imply that the flexibility of *Region II* residues of PH domain may correlate the size of binding group.

The *Region III* is located on the solvent-accessible side of the β_7_, especially I92_GAB1_ or H92_IRS1_ (β_7_[3]) (**Fig. 7A–E**). When GAB-010 binds IRS1 PH domain, the benzimidazole moiety induced a significant side chain movement of H92 (RMSD = 3.39 Å) as compared with unbound form (magenta plots on the right in **Fig. 7A** and **Movie S3** available at http://imdlab.org/supporting/PLOSCompBio). In contrast, this region in GAB1 PH domain did not exhibit significant conformational changes when binding any inhibitor (magenta plots on the left in **Fig. 7A**). Upon comparison of GAB1 and IRS1 PH domain sequences, we speculated that the accessibility of *Region III* was affected by the length of β_1,2_ loop: GAB1 PH domain has a longer β_1,2_ loop than IRS1 (**Fig. 2**), and the residues P16 and P17 forms intensive vdW interactions with β_7_ (**Fig. 2C**), which would in turn block the access of inhibitors to *Region III*. This explains the selective binding of GAB-010 to IRS1, but not GAB1 PH domain.

## Discussion

GAB1 is a critical protein in cellular signaling, and its PH domain has been suggested as an attractive target for various cancer treatments [34], [35]. However, the absence of its 3D structure makes it challenging for structure-based drug discovery. We herein present a rigorously designed workflow for inhibitor identification by integrating various techniques ranging from structural bioinformatics, homology modeling, ligand-steered refinement, molecular dynamics, and virtual screening, followed by experiment evaluation with biochemical/biophysical and cellular assays. With our integrated protocol, we have successfully identified several selective inhibitors targeting the GAB1 PH domain and they are selective to breast cancer cells. This discovery offers us a great starting point to target this critical protein for cancer treatment, particularly for the triple negative breast cancer.

Our results also showed that the triple-negative breast cancer cell line, MDA-MB-231, was more resistant to GAB1 inhibitors than ER-positive breast cancer cell line, T47D (**Table 1**). It has been reported that MDA-MB-231, but not T47D, has mutations on GAB1 downstream proteins, such as KRas and BRaf mutations [36]. Since KRas and BRaf mutations are known to reduce the dependency on the upstream activators, such as EGFR [37], it was not surprising that MDA-MB-231 was more resistance to GAB1 inhibitors. Strikingly, we observed a concomitant inhibition of pGAB1 and pIRS1 by either GAB1-specific or IRS1-specific inhibitors (**S7 Fig.**). This could be due to the crosstalk between c-Met and α6β4 integrin pathway [29], which couples the phosphorylation of GAB1 and IRS1 upon HGF stimulation. These observations may bring us new insights of combined PH domain-targeted cancer therapeutic strategies. Further mechanistic studies are ongoing to investigate these hypotheses.

While it is exciting to see that we have identified selective inhibitors of the GAB1 PH domain using our unique computation-experimentation integrated platform, we have to admit that some of the other hits also bind to multiple PH domains (e.g., IRS1 and AKT1), as demonstrated by **Table 1**. For example, GAB-001 and GAB-004 are selectively inhibitor GAB1, but GAB-016 and GAB-017 are pan inhibitors against GAB1, IRS1 and AKT1. More follow-up experiments also showed that GAB-016 targets GAB2 PH domain as well. This is not surprising because PH domain is defined by their common β-sandwich structure. In addition, GAB1 and GAB2 PH domains are highly homologous (76% sequence identity), and IRS1 is one of the templates used in our homology modeling to build the 3D structure of GAB1 PH domain. Of note, all GAB1-selective or IRS1-selective inhibitors showed much better IC_50_ against T47D and MDA-MB-231 breast cancer cell lines than the non-tumorigenic MCF-10A cell line (**Fig. 5**). More intriguingly, we also observed that AKT1-selective inhibitors (e.g., GAB-012 and GAB-013) were toxic to MCF-10A at 100 µM, but not for T47D and MDA-MB-231 at the same concentration (data not shown). This may imply that targeting GAB1 or IRS1, but not AKT1, might be a better targeted strategy for breast cancer treatment.

Although PH domains have been intensively studied as cancer target for drug discovery, to date there are no available protein structures in complex with any drug-like small molecules. As mentioned, this has significantly limited the structure-based drug discovery efforts. In the present work, we utilized several inhibitors to investigate the dynamics of GAB1 PH domain and evaluate their selectivity in potential cancer cell inhibition. Interestingly, we found that the *apo*-structure of the PH domain protein could undergo large conformational changes in three regions to accommodate different inhibitors. The side-chain conformations of the residues in *Region I* determines the binding of either multiple electronegative groups (e.g., the multiple phosphates in IP4) or an aromatic moiety conjugated with a group containing H-bond acceptors (e.g., benzenesulfone), as shown in **Fig. 7**. The accessibility of *Region II* and *Region III* depend on several critical amino acids on β_4_ and β_7_ and the length of β_1,2_ loop, respectively. The selectivity of PH domain inhibitors may be designed based on our modeling of the protein structures. For instance, GAB-010 is highly selective to IRS1 but no binding to GAB1 or AKT1, largely due to the short β_1,2_ loop. The knowledge that GAB1 PH domain undergoes conformational change upon ligand binding provides novel insights of guiding the future structure-based drug design efforts, and of course more experimental validation will increase our understanding of GAB1 structure and functions.

## Materials and Methods

### Chemical dataset

A collection of five million drug and lead-like compounds which were curated from various sources (e.g., PubChem [38] and MayBridge) was used for virtual screening. LigPrep [39] was employed for ligand preparation, including the removal of salts, assignment of appropriate protonation, tautomerization and ring conformations, and generation of 3D structures by energy minimization with OPLS2001 force field [40]. Additionally, an internal collection of 167 previously synthesized inhibitors targeting AKT PH domain were included for virtual screening.

### PSSM and sequence logo representation

A total of 65 high-resolution crystal structures of PH domains were obtained from PDB, and we curated 34 non-redundant proteins. Their PDB IDs are listed in **S2 Table**. They were used for secondary structure-based sequence alignment with STRAP [15]. We extracted the multiple sequence alignments for β_1_, β_2_, β_3_, β_6_, β_7_ and α_1_ secondary structural fragments. The individual alignment will be used as input to PSI-BLAST [41] which could generate a PSSM for each individual fragment as shown in **S3 Table**. These PSSMs can be represented by WebLogo for more intuitive visualization and understanding (**S1 Fig.**). These figures were generated using the WebLogo server [42].

### 3D structure prediction of GAB1 PH domain in complex with IP4

The sequence of GAB1 PH domain was retrieved from UniProt database (accession number Q13480) [43]. The secondary structure was predicted by PSSM combined with PSIPRED [16] and aligned to the templates (myosin X (PDB ID: 3TFM [17]), IRS1 (PDB ID: 1QQG [18]), TAPP1 (PDB ID: 1EAZ [19]) and DAPP1 (PDB ID: 1FAO [20]) for homology modeling. To improve the quality of homology modeling, we manually corrected the multiple sequence alignment generated by ClustalX to ensure each secondary structure elements (e.g., α-helix and β-sheets) were properly aligned. GAB1 PH domain homology models were built using MODELLER 9v10 [44]. As the active site residues of DAPP1 have the highest homology to those of GAB1 PH domain, the coordinates of the IP4 co-crystallized with DAPP1 was used as the initial structure. We generated initial 1,000 homology models. Since the lysine-rich loop β_1,2_ is important for phosphoinositide binding, especially for Group 1 PH domain [7], [45], the β_1,2_ loop of top ten initial models (evaluated by DOPE score) were subjected to ligand-steered refinement using built-in function of MODELLER. We selected five models out of the 100 generated loop models based on the overall DOPE scores [46], Ramachandran plot, and the consistencies to IP4 binding site features [24], [25], [47]–[51] and the reported mutagenesis studies [6], [8]. These five GAB1-IP4 complex models were refined by MD simulations using AMBER10 available at Texas Advanced Computing Center. All MD simulations were performed in triplicates with different initial velocities. The MD simulations were performed using ff99SB force field [32] in TIP3P explicit solvent with particle mesh Ewald (PME), periodic boundary conditions and SHAKE. The topology and charges of the ligand were generated by Antechamber with AM1-BCC charges [52]. The system is solvated and neutralized in the cuboid box in which the closest distance between any atom originally in solute and the edge of the box is 12 Å. The system was equilibrated for 100 ps, and the production MD simulations were run in NPT ensemble for 20 ns, with the time step = 2 fs. The snapshots were taken every 1 ps. The root mean square deviation (RMSD) relative to the first frame and the root mean square fluctuation (RMSF) relative to the average structure were analyzed with cpptraj implemented in AmberTools12 [52]. The average structures were minimized, and the model quality was evaluated by QMEAN [21], ProSA [22] and PROCHECK [23]. A reasonable protein model should have both ProSA and QMEAN Z-scores within the range for the native proteins of similar size, as illustrated by **S3 Fig**.

### Virtual screening

GOLD 5.1 [53] was employed for virtual screening on our high performance computing cluster using the GAB1-IP4 complex model derived above. Molecular docking was performed with flexible side chains of the residues involved in IP4 binding, and the conformation with the best score of each compound was ranked based on their ChemPLP scores. Protein pharmacophore modeling was performed using GRID v22c [54]. Briefly, the GRID calculations were performed with a grid box enclosing the target with 1 Å beyond each dimension. During the calculations, the GRID directive Move was set (MOVE = 1) to allow the flexibility of the side chains. The molecular interaction fields (MIFs) [55] were computed to determine the interaction between the receptor atoms and three different probes: the hydrophobic (DRY), the amide nitrogen (N1, H bond donor), and the carbonyl oxygen (O, H bond acceptor). Via visual inspection of the local minima of the GRID energy maps, the favorable binding sites of these three probes were used to define the features of a pharmacophore query. The derived pharmacophores were used to evaluate the binding poses of the initially selected 10,000 hits out of the five million compounds. If the docked hit poses fit the pharmacophore, they would be selected and subjected to clustering analysis based on the MACCS fingerprints and Tanimoto coefficient. The best scored compound from each cluster was chosen and the binding poses of these hits were individually inspected based on molecular visualization.

### 3D structure refinement of GAB1/IRS1 PH domain in complex with inhibitors

In order to evaluate the selectivity of our inhibitors, we optimized the also PH domain (IRS1 or GAB1) -inhibitor complex structures using MD simulations. The starting conformation for MD simulation is the binding mode which obtained the best score in molecular docking. The MD simulations were performed in triplicates for 50 ns using the parameters described in “3D structure prediction of GAB1 PH domain in complex with IP4” section. We also generated the trajectory of GAB1-GAB-001 complex (**Movie S1**), GAB1-GAB-017 (**Movie S2**) and IRS1-GAB-010 (**Movie S3**). Each trajectory contained 1,000 snapshots which were taken every 50 ps. The ligands and the critical residues were in sticks, whereas the backbones of PH domain proteins were in ribbons. Starting from the docking conformation, these MD trajectories vividly demonstrated the conformational changes of the PH domain proteins upon ligand binding. The movies were available at http://imdlab.org/supporting/PLOSCompBio.

### PMF-based binding free energy calculation

The routine of PMF-based computation of protein-ligand absolute binding free energy has been previously described [30], [31]. Briefly, the average structure of protein-ligand complex obtained from three independent 50 ns MD simulations was subject to energy minimization to remove clashes. The resulted structure was considered as the reference frame to define the position and orientation constraints. The PMF as a function of mass-weighted RMSD (*ξ*) relative to the reference ligand or the protein-ligand distance (*r*) was sampled by umbrella sampling and weighted-histogram analysis method (WHAM). The full description of this method is available in **S1 Method**. The experimental binding free energies were derived from experimental *K*_D_ (or *K*_i_) using the equation 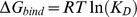 or 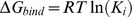.

### Surface plasmon resonance (SPR) spectroscopy binding assays

The DNA sequences of human GAB1 and IRS1 PH domain (IRS1 is for selectivity evaluation) were cloned into pGEX-4T1 inducible bacterial expression plasmid (GeneStorm, Invitrogen, Carlsbad, CA) transformed into BL21 (DE3) *E. Coli*. Expression and purification of the recombinant proteins were performed as previously described [51]. Binding assays were performed using a Biacore 2000 instrument with the Biacore Control Software v3.2 and BIAevaluation v4.1 analysis software (Biacore, Piscataway, NJ) as previously described [51]. Briefly, the PH domain GST-fusion proteins were immobilized on a CM5 Sensorchip (Biacore BR-1000-12) using Biacore's Amine Coupling Kit (Biacore BR-1000-50) to a level of 10,000 Response units (RUs). Small molecule analytes at concentrations ranging from one tenth to ten times the predicted *K*_D_ were injected at a high flow rate (30 µL/min). Dimethylsulfoxide (DMSO) concentrations in all samples and running buffer were 1% (v/v) or less. For the competitive binding assays and *K*_i_ determination, PtdIns(3,4,5)P_3_-biotin labeled liposomes (Echelon Biosciences, Salt Lake City, UT) and SA chips were used with increasing concentrations of the compound tested. We did triplicate SPR assays for each concentration.

### Cellular proliferation assay

Two human breast cancer cell lines and one normal breast cell line were used for this study: T47D ductal breast epithelial tumor cell line, MDA-MB-231 epithelial tumor cell line and MCF-10A non-tumorigenic epithelial cell line (American Type Culture Collection, Rockville, MD). T47D and MDA-MB-231 cells were maintained in bulk culture in Dulbecco's modified Eagle medium (DMEM) supplemented with 10% heat-inactivated fetal bovine serum (FBS), 4.5 g/L glucose, 100 U/mL penicillin and 100 mg/mL streptomycin in a 5% CO_2_ atmosphere. MCF-10A cells were maintained in MEGM with other conditions same as the cancer cell lines. Cells were passaged using 0.25% trypsin and 0.02% EDTA. Cells were confirmed to be mycoplasma free by testing them with an ELISA kit (Roche-Boehringer Mannheim, Indianapolis, IN). Our hit compounds were freshly prepared in DMSO at a stock concentration of 10 mM. For the evaluation of cellular proliferation, a standard 96-well micro-cytotoxicity assay was performed as described in reference [51]. Briefly, the assay was set up by plating cells at 5,000–10,000 cells per well (depending on cell doubling time) for a growth period of 4 days. The identified hits were added directly to the media, dissolved in DMSO at various concentrations ranging from 1 to 200 µM. The endpoint was spectrophotometric determination of the reduction of 3-(4,5-dimethylthiazol-2-yl)-2,5-diphenyltetrazolium bromide. All assays were performed in triplicates.

### Inhibition of GAB1 and IRS1 phosphorylation

For all biological assays, hit compounds were added at 20 µM concentration directly into the culture media of the cells for 4 hr following a 16 hr incubation of T47D cells without FBS. Cells were stimulated with HGF for 20 min at 50 ng/ml. Following this treatment, cells were lysed as previously published [51] and equal amounts of total cell lysate were loaded on a pSer^312^-IRS-1/Total IRS-1 Meso Scale Discovery plate as described by the manufacturer. The plate was read using a Sector Imager 2400A instrument (Meso Scale Discovery protein profiling system, Gaithersburg, MD). For the measurement of GAB1 phosphorylation, T47D cells were treated as for the phosphorylation of IRS1 evaluation. Cell lysates were run on a 7% SDS-PAGE and membrane were probed with specific anti-phospho-Tyr^627^ GAB1 (Cell signaling). Each experiment was performed at least three times.

## Supporting Information

S1 Fig **Sequence logos for PH domain.** Position-specific logos for the secondary structure elements of PH domain (generated by Weblogo server). β_4_ and β_5_ are not included because of the high variation within these two β-sheets. The size of residue indicates the relative frequency of the residue at the corresponding position.
(PDF)

S2 Fig **Backbone RMSD and per-residue backbone RMSF for GAB1-IP4 complex.** The simulation lasted for 20 ns. The mass-weighted average RMSFs were calculated for each residue based on backbone atoms. Atomic RMSFs of four phosphates in IP4 were shown in the last table.
(PDF)

S3 Fig **Quality of GAB1 PH domain model.** This model was subjected to energy minimization prior to quality assessment. (A) QMEAN; (B) ProSA; (C); Ramachandran plot and summary generated by PROCHECK.
(PDF)

S4 Fig **Comparison of DOPE score profiles.** IRS1 (PDB ID: 1QQG), GAB1 homology model and the lowest energy GAB1 model with MD simulation refinement were compared. The MD refinement model was subject to 1000 steps energy minimization with ff99SB force field to remove transient structural defects. The secondary structure is annotated at the bottom.
(PDF)

S5 Fig **Isovolume of GAB1 PH domain generated by GRID.** The area favoring H-bond acceptor (−6.0 kcal/mol) was displayed in magenta surface, whereas area favoring hydrophobic (−2.0 kcal/mol) probes was displayed in green surface. The protein backbone is illustrated with cyan lines, and the ligand (IP4) is in blue sticks. The crucial residues are labeled around isovolume surface.
(PDF)

S6 Fig **SPR of GAB-001, GAB-016 binding to GAB1 PH domain.**
(PDF)

S7 Fig **Inhibition of S312 phosphorylation of IRS1.** The percentage of inhibition values are calculated from three separated experiments.
(PDF)

S8 Fig **Backbone RMSD of GAB1PH/IRS1PH – inhibitor complex.**
(PDF)

S9 Fig **Schematic representation of frame of reference used to define the position and orientation restraints.** To reduce the degrees of freedom, P1, P2, P3 and L1, L2, L3 were employed to represent the coordinates of the protein and the ligand, respectively, as described in **S1 Method**.
(PDF)

S10 Fig **Ligand conformational PMF as a function of mass-weighted RMSD (ξ).** The ligand in the bulk is in black lines, whereas the ligand in the active site is in read lines. (**A–E**). PMFs derived from GAB1-inhibitor complexes. (**F–H**). PMFs derived from IRS1-inhibitor complexes.
(PDF)

S11 Fig **Separation PMF as a function of L1-P1 distance (r).** (**A–E**). PMFs derived from GAB1-inhibitor complexes. (**F–H**). PMFs derived from IRS1-inhibitor complexes.
(PDF)

S12 Fig **Pharmacophore of the inhibitors.** Green: hydrophobic or aromatic region. Blue: the projection of H-bond acceptor.
(PDF)

S1 Table **Docking scores for 20 htis.** The hits which are consistently active in three assays are labeled with bold IDs.
(PDF)

S2 Table **The crystal structures employed to generate PSSM.**
(PDF)

S3 Table **PSSM profile for PH domain (β_1_, β_2_, β_3_, β_6_, β_7_ and α_1_).**
(PDF)

S1 Method **Technical details for computational modeling.**
(PDF)
