# Supplementary material for: Novel Inhibitors Induce Large Conformational Changes of GAB1 Pleckstrin Homology Domain and Kill Breast Cancer Cells
Source: PLoS Comput Biol. 2015 Jan 8;11(1):e1004021. doi: 10.1371/journal.pcbi.1004021 (PMC4287437; doi:10.1371/journal.pcbi.1004021)

Figure S1. **Sequence logos for PH domain.** Position-specific logos for the secondary structure elements of PH domain (generated by Weblogo server). β_4 and β_5 are not included because of the high variation within these two β-sheets. The size of residue indicates the relative frequency of the residue at the corresponding position.

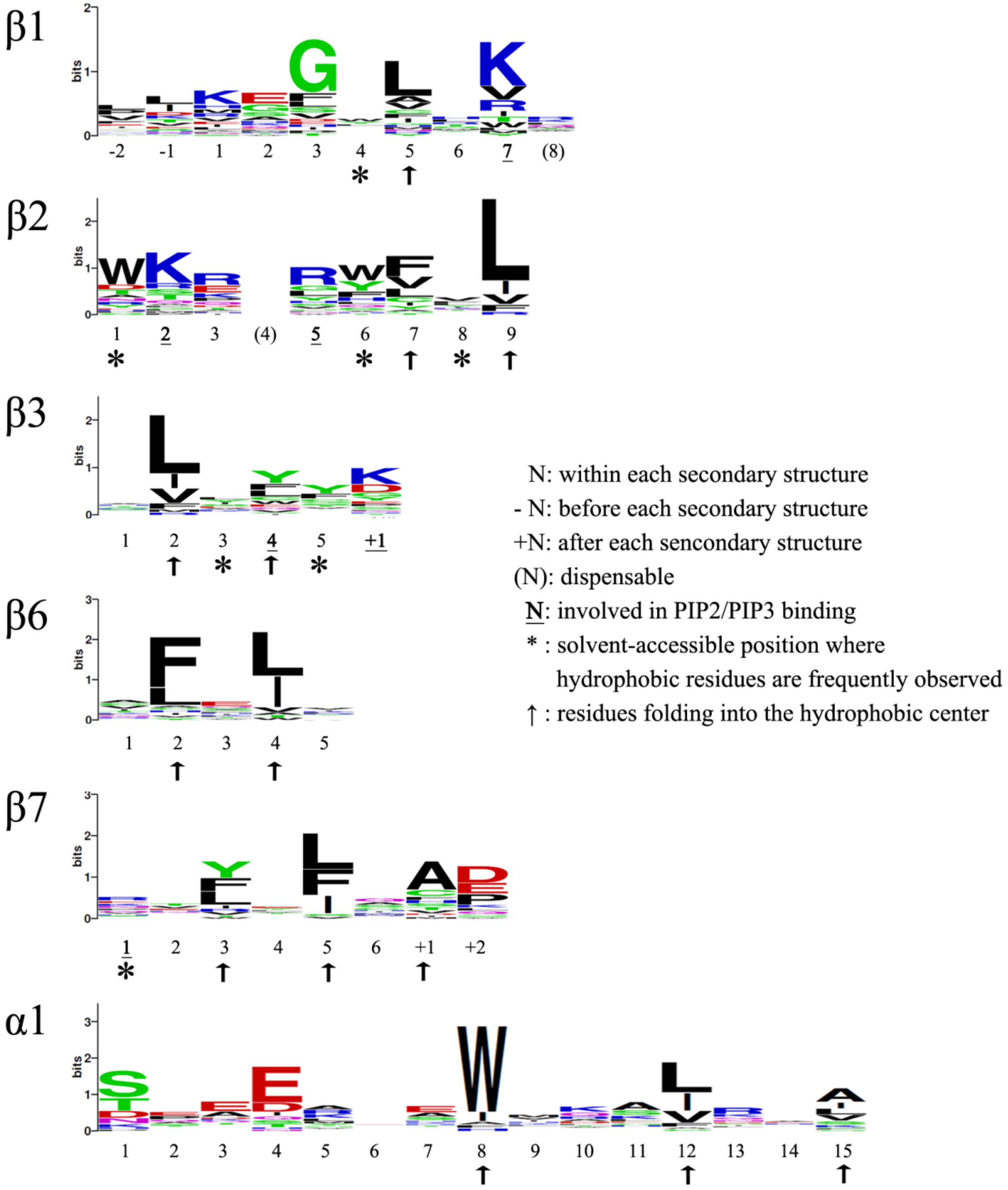

Supplement: S1 Fig — Sequence logos for PH domain. Position-specific logos for the secondary structure elements of PH domain (generated by Weblogo server). β4 and β5 are not included because of the high variation within these two β-sheets. The size of residue indicates the relative frequency of the residue at the corresponding position. (PDF) [file pcbi.1004021.s001.pdf]
