# Supplementary material for: Novel Inhibitors Induce Large Conformational Changes of GAB1 Pleckstrin Homology Domain and Kill Breast Cancer Cells
Source: PLoS Comput Biol. 2015 Jan 8;11(1):e1004021. doi: 10.1371/journal.pcbi.1004021 (PMC4287437; doi:10.1371/journal.pcbi.1004021)
Supplement: S2 Fig — Backbone RMSD and per-residue backbone RMSF for GAB1-IP4 complex. The simulation lasted for 20 ns. The mass-weighted average RMSFs were calculated for each residue based on backbone atoms. Atomic RMSFs of four phosphates in IP4 were shown in the last table. (PDF) [file pcbi.1004021.s002.pdf]

Figure S2. Backbone RMSD and per-residue backbone RMSF for GAB1-IP4 complex. The simulation lasted for 20ns. The mass-weighted average RMSFs were calculated for each residue based on backbone atoms. Atomic RMSFs of four phosphates in IP4 were shown in the last table.

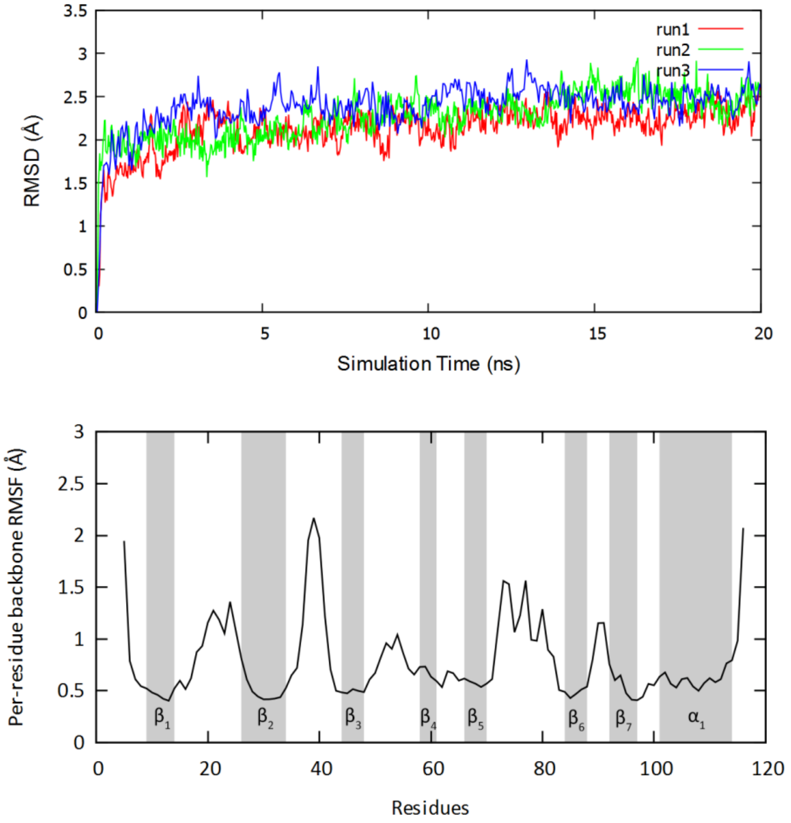

Structure	Position	RMSF (Å)
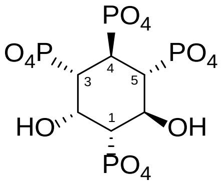	1-P	0.79
	3-P	0.62
	4-P	0.83
	5-P	1.02
