# Supplementary material for: Novel Inhibitors Induce Large Conformational Changes of GAB1 Pleckstrin Homology Domain and Kill Breast Cancer Cells
Source: PLoS Comput Biol. 2015 Jan 8;11(1):e1004021. doi: 10.1371/journal.pcbi.1004021 (PMC4287437; doi:10.1371/journal.pcbi.1004021)
Supplement: S3 Fig — Quality of GAB1 PH domain model. This model was subjected to energy minimization prior to quality assessment. (A) QMEAN; (B) ProSA; (C); Ramachandran plot and summary generated by PROCHECK. (PDF) [file pcbi.1004021.s003.pdf]

Figure S3. Quality of GAB1 PH domain model. This model was subjected to energy minimization prior to quality assessment. (A) QMEAN; (B) ProSA; (C); Ramachandran plot and summary generated by PROCHECK.

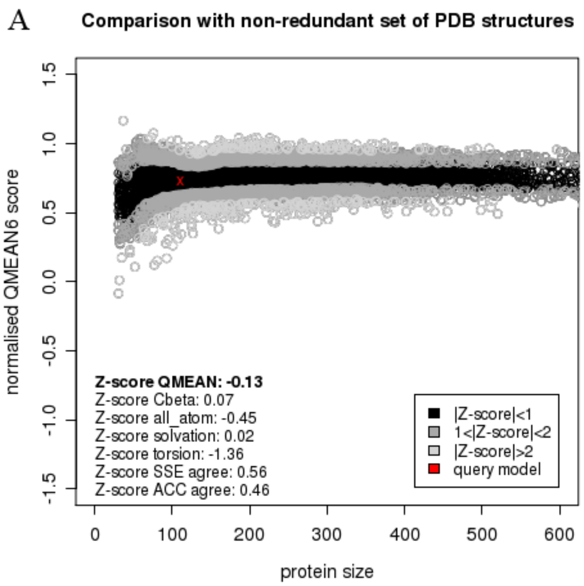

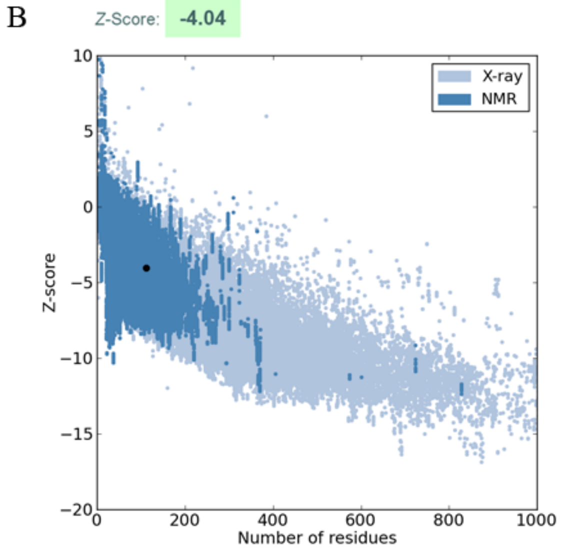

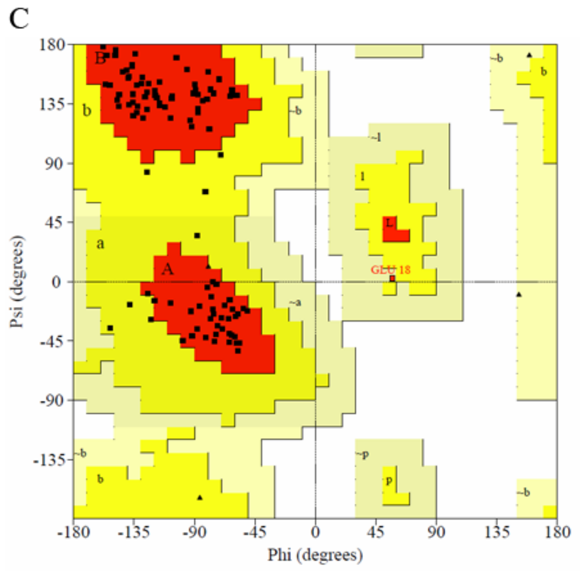

Plot statistics

Residues in most favoured regions [A,B,L]	94	92.2%
Residues in additional allowed regions [a,b,l,p]	7	6.9%
Residues in generously allowed regions [~a,~b,~l,~p]	1	1.0%
Residues in disallowed regions	0	0.0%
	----	------
Number of non-glycine and non-proline residues	102	100.0%
Number of end-residues (excl. Gly and Pro)	1	
Number of glycine residues (shown as triangles)	5	
Number of proline residues	4	

Total number of residues	112	

Based on an analysis of 118 structures of resolution of at least 2.0 Angstroms and R-factor no greater than 20%, a good quality model would be expected to have over 90% in the most favoured regions.
