# Supplementary material for: Novel Inhibitors Induce Large Conformational Changes of GAB1 Pleckstrin Homology Domain and Kill Breast Cancer Cells
Source: PLoS Comput Biol. 2015 Jan 8;11(1):e1004021. doi: 10.1371/journal.pcbi.1004021 (PMC4287437; doi:10.1371/journal.pcbi.1004021)

Figure S4. **Comparison of DOPE score profiles.** IRS1 (PDB ID: 1QQG), GAB1 homology model and the lowest energy GAB1 model with MD simulation refinement were compared. The MD refinement model was subject to 1000 steps energy minimization with ff99SB force field to remove transient structural defects. The secondary structure is annotated at the bottom.

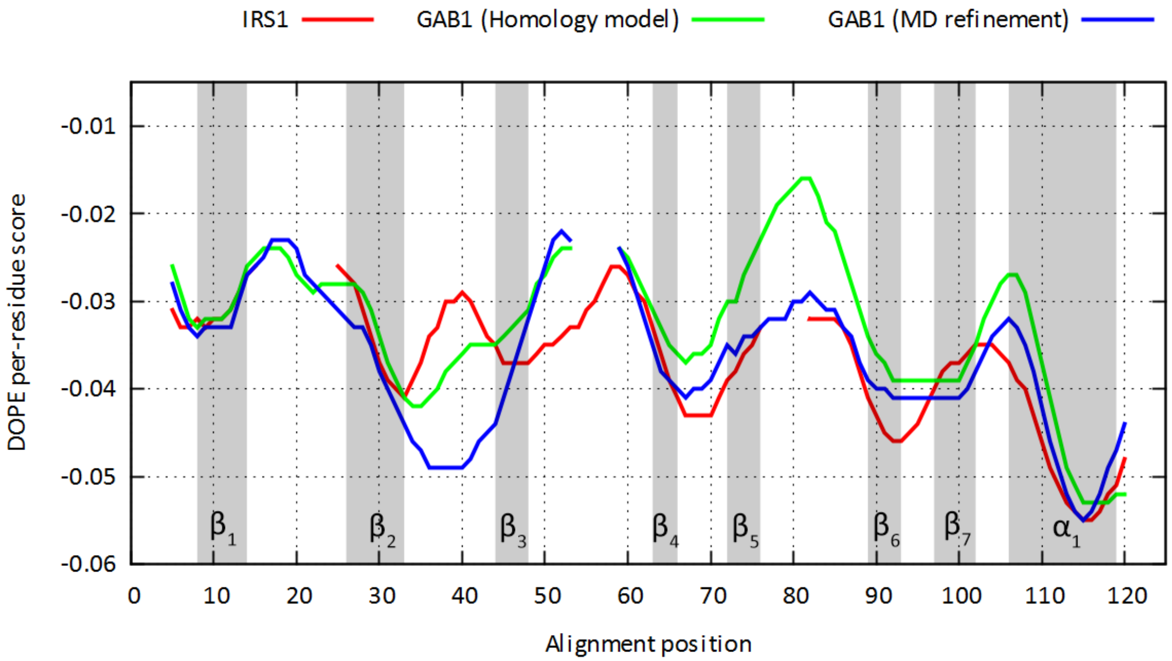

Supplement: S4 Fig — Comparison of DOPE score profiles. IRS1 (PDB ID: 1QQG), GAB1 homology model and the lowest energy GAB1 model with MD simulation refinement were compared. The MD refinement model was subject to 1000 steps energy minimization with ff99SB force field to remove transient structural defects. The secondary structure is annotated at the bottom. (PDF) [file pcbi.1004021.s004.pdf]
