# Supplementary material for: Novel Inhibitors Induce Large Conformational Changes of GAB1 Pleckstrin Homology Domain and Kill Breast Cancer Cells
Source: PLoS Comput Biol. 2015 Jan 8;11(1):e1004021. doi: 10.1371/journal.pcbi.1004021 (PMC4287437; doi:10.1371/journal.pcbi.1004021)

Figure S5. **Isovolume of GAB1 PH domain generated by GRID.** The area favoring H-bond acceptor (-6.0 kcal/mol) was displayed in magenta surface, whereas area favoring hydrophobic (-2.0 kcal/mol) probes was displayed in green surface. The protein backbone is illustrated with cyan lines, and the ligand (IP4) is in blue sticks. The crucial residues are labeled around isovolume surface.

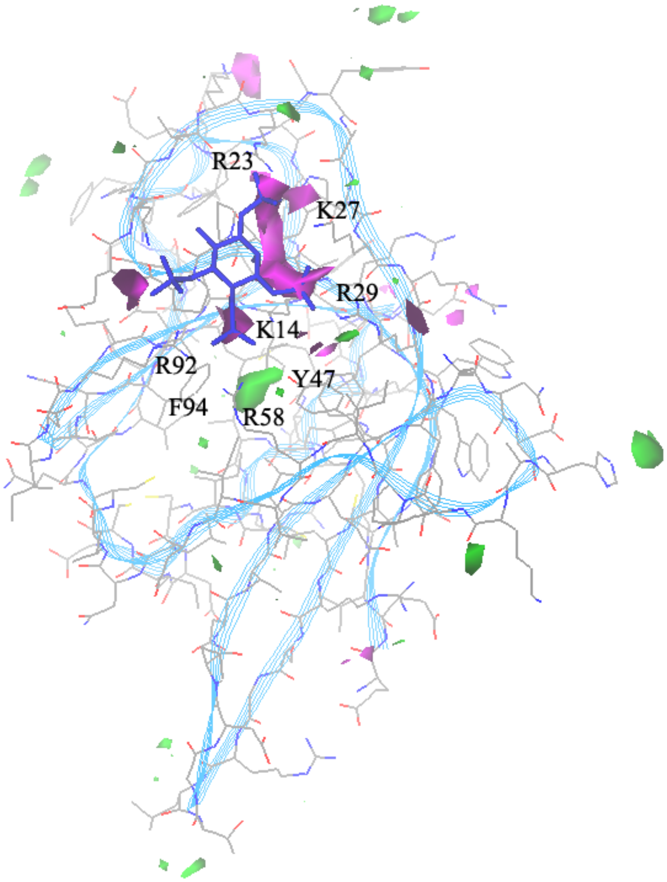

Supplement: S5 Fig — Isovolume of GAB1 PH domain generated by GRID. The area favoring H-bond acceptor (−6.0 kcal/mol) was displayed in magenta surface, whereas area favoring hydrophobic (−2.0 kcal/mol) probes was displayed in green surface. The protein backbone is illustrated with cyan lines, and the ligand (IP4) is in blue sticks. The crucial residues are labeled around isovolume surface. (PDF) [file pcbi.1004021.s005.pdf]
