# Supplementary figures and images for: Novel Inhibitors Induce Large Conformational Changes of GAB1 Pleckstrin Homology Domain and Kill Breast Cancer Cells
Source: PLoS Comput Biol. 2015 Jan 8;11(1):e1004021. doi: 10.1371/journal.pcbi.1004021 (PMC4287437; doi:10.1371/journal.pcbi.1004021)

**Figure S6. SPR of GAB-001, GAB-016 binding to GAB1 PH domain.**

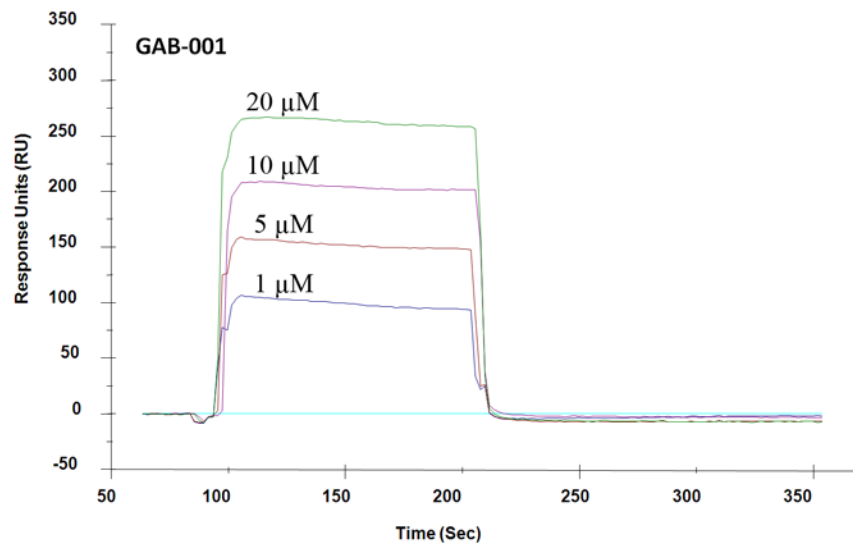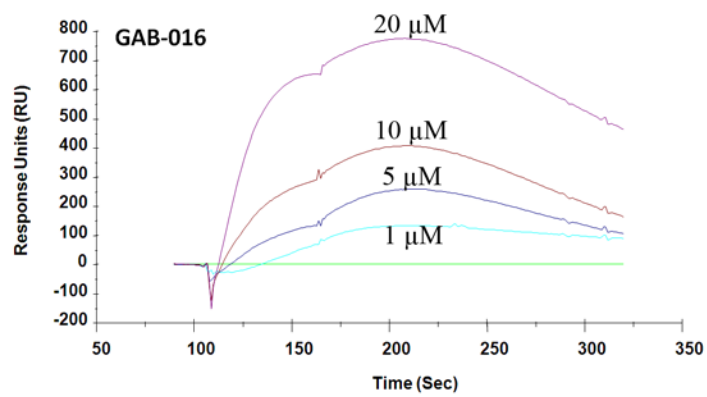

Supplement: S6 Fig — SPR of GAB-001, GAB-016 binding to GAB1 PH domain. (PDF) [file pcbi.1004021.s006.pdf]

**Figure S8. Backbone RMSD of GAB1PH/IRS1PH – inhibitor complex.**

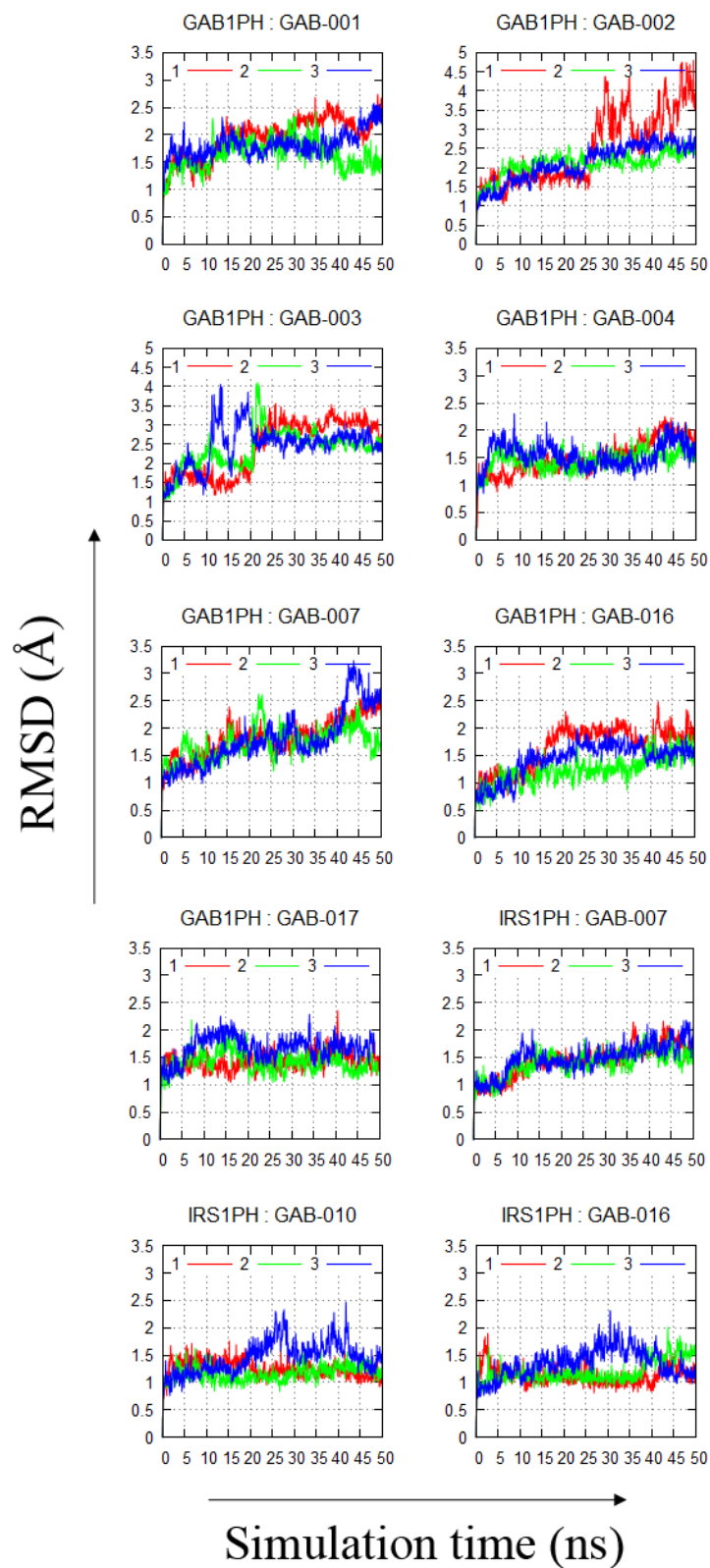

Supplement: S8 Fig — Backbone RMSD of GAB1PH/IRS1PH – inhibitor complex. (PDF) [file pcbi.1004021.s008.pdf]
