# Supplementary material for: Novel Inhibitors Induce Large Conformational Changes of GAB1 Pleckstrin Homology Domain and Kill Breast Cancer Cells
Source: PLoS Comput Biol. 2015 Jan 8;11(1):e1004021. doi: 10.1371/journal.pcbi.1004021 (PMC4287437; doi:10.1371/journal.pcbi.1004021)
Supplement: S7 Fig — Inhibition of S312 phosphorylation of IRS1. The percentage of inhibition values are calculated from three separated experiments. (PDF) [file pcbi.1004021.s007.pdf]

Figure S7. Inhibition of S312 phosphorylation of IRS1. The percentage of inhibition values are calculated from three separated experiments.

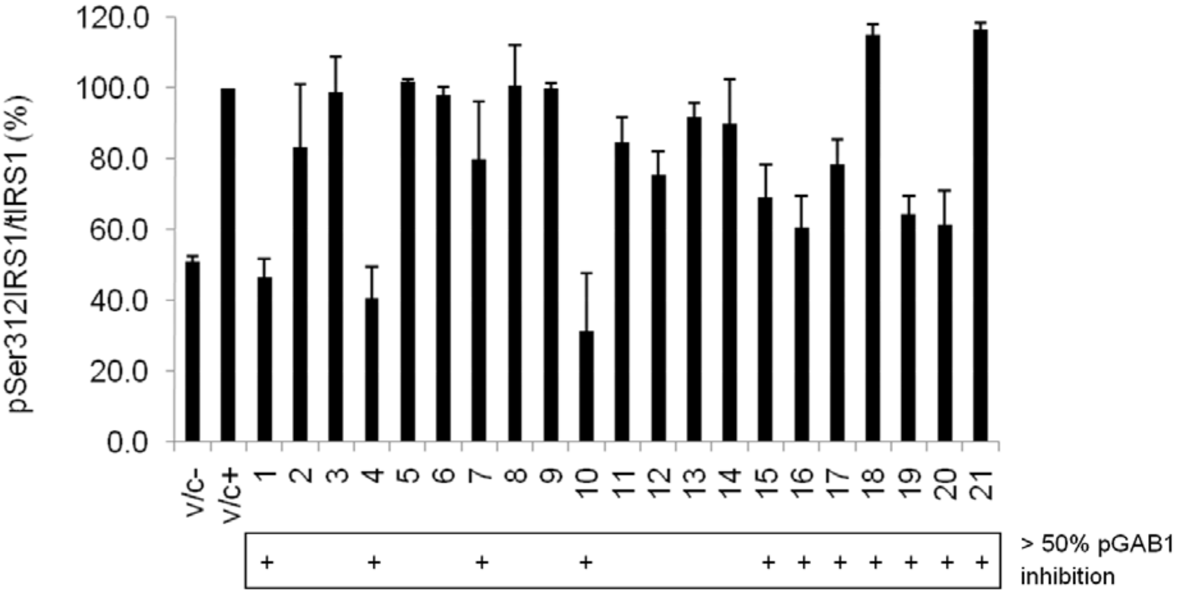

Raw data

	V/C-	V/C+	1	2	3	4	5	6	7	8	9	10
Avg	50.8	100.0	46.5	83.2	98.7	40.7	101.8	98.2	79.7	100.7	100.0	31.1
StdDev	1.56	0.02	5.40	17.82	10.08	8.93	0.83	1.92	16.65	11.56	1.38	16.63

	11	12	13	14	15	16	17	18	19	20	21
Avg	84.8	75.6	91.7	89.9	69.0	60.6	78.4	115.3	64.4	61.3	116.5
StdDev	6.79	6.36	4.06	12.61	9.46	8.82	7.07	2.70	5.00	9.61	2.09
