# Supplementary material for: Novel Inhibitors Induce Large Conformational Changes of GAB1 Pleckstrin Homology Domain and Kill Breast Cancer Cells
Source: PLoS Comput Biol. 2015 Jan 8;11(1):e1004021. doi: 10.1371/journal.pcbi.1004021 (PMC4287437; doi:10.1371/journal.pcbi.1004021)

Figure S9. **Schematic representation of frame of reference used to define the position and orientation restraints.** To reduce the degrees of freedom, P1, P2, P3 and L1, L2, L3 were employed to represent the coordinates of the protein and the ligand, respectively, as described in **Method S1**.

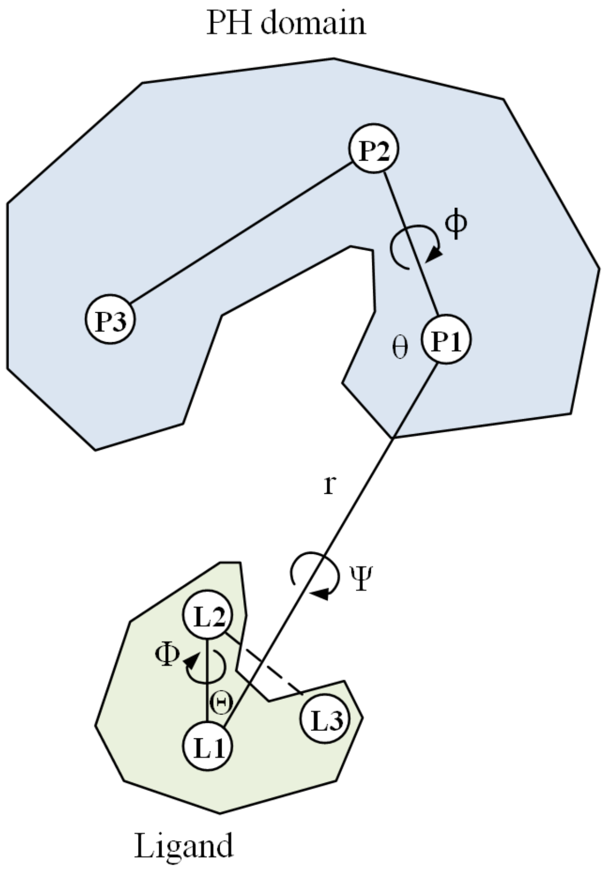

Supplement: S9 Fig — Schematic representation of frame of reference used to define the position and orientation restraints. To reduce the degrees of freedom, P1, P2, P3 and L1, L2, L3 were employed to represent the coordinates of the protein and the ligand, respectively, as described in S1 Method . (PDF) [file pcbi.1004021.s009.pdf]
