# Supplementary material for: Novel Inhibitors Induce Large Conformational Changes of GAB1 Pleckstrin Homology Domain and Kill Breast Cancer Cells
Source: PLoS Comput Biol. 2015 Jan 8;11(1):e1004021. doi: 10.1371/journal.pcbi.1004021 (PMC4287437; doi:10.1371/journal.pcbi.1004021)

Figure S10. Ligand conformational PMF as a function of mass-weighted RMSD (ξ). The ligand in the bulk is in black lines, whereas the ligand in the active site is in read lines. (**A-E**). PMFs derived from GAB1-inhibitor complexes. (**F-H**). PMFs derived from IRS1-inhibitor complexes.

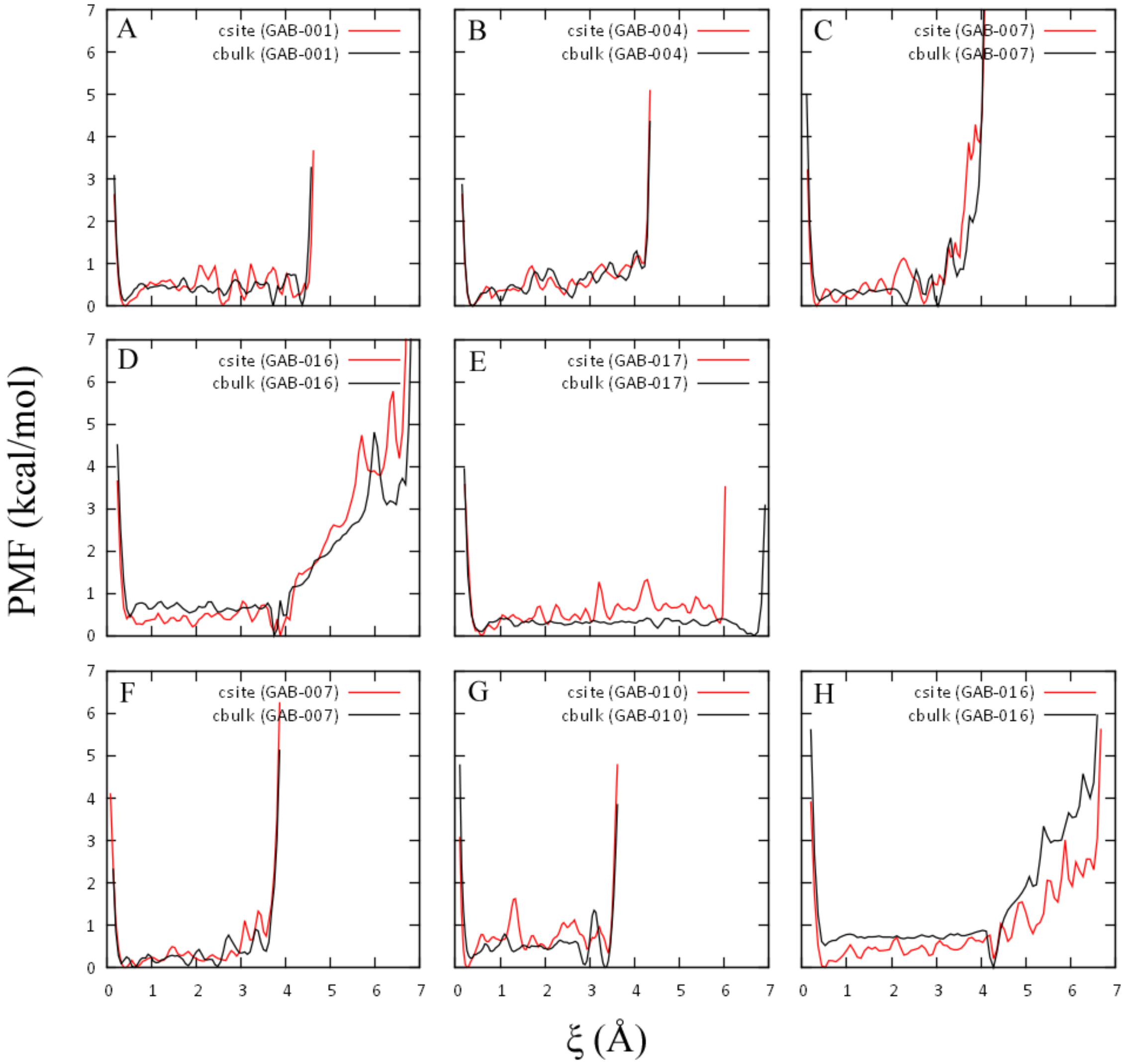

Supplement: S10 Fig — Ligand conformational PMF as a function of mass-weighted RMSD (ξ). The ligand in the bulk is in black lines, whereas the ligand in the active site is in read lines. (A–E). PMFs derived from GAB1-inhibitor complexes. (F–H). PMFs derived from IRS1-inhibitor complexes. (PDF) [file pcbi.1004021.s010.pdf]
