# Supplementary material for: Novel Inhibitors Induce Large Conformational Changes of GAB1 Pleckstrin Homology Domain and Kill Breast Cancer Cells
Source: PLoS Comput Biol. 2015 Jan 8;11(1):e1004021. doi: 10.1371/journal.pcbi.1004021 (PMC4287437; doi:10.1371/journal.pcbi.1004021)

Figure S11. **Separation PMF as a function of L1-P1 distance (r).** (**A-E**). PMFs derived from GAB1-inhibitor complexes. (**F-H**). PMFs derived from IRS1-inhibitor complexes.

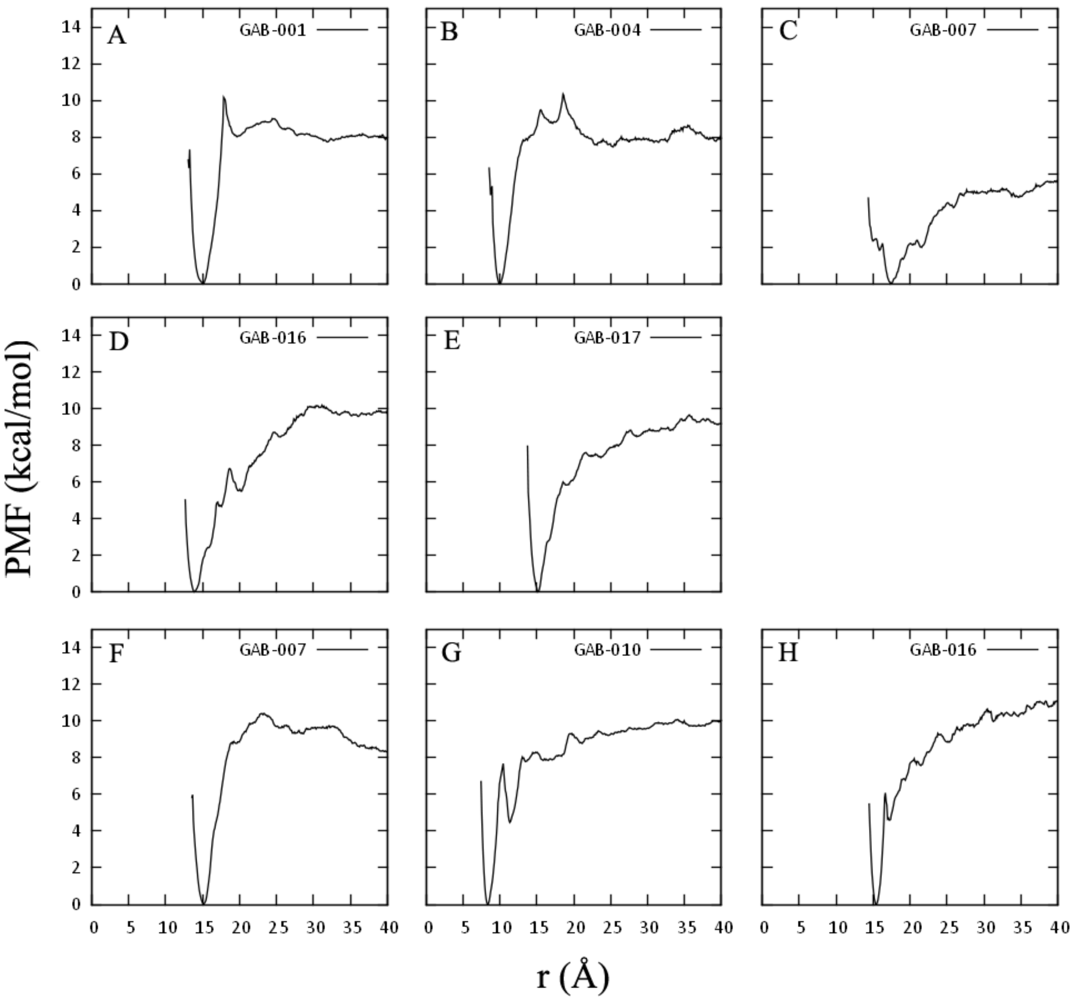

Supplement: S11 Fig — Separation PMF as a function of L1-P1 distance (r). (A–E). PMFs derived from GAB1-inhibitor complexes. (F–H). PMFs derived from IRS1-inhibitor complexes. (PDF) [file pcbi.1004021.s011.pdf]
