# Supplementary material for: Novel Inhibitors Induce Large Conformational Changes of GAB1 Pleckstrin Homology Domain and Kill Breast Cancer Cells
Source: PLoS Comput Biol. 2015 Jan 8;11(1):e1004021. doi: 10.1371/journal.pcbi.1004021 (PMC4287437; doi:10.1371/journal.pcbi.1004021)

Figure S12. **Pharmacophore of the inhibitors.** Green: hydrophobic or aromatic region. Blue: the projection of H-bond acceptor.

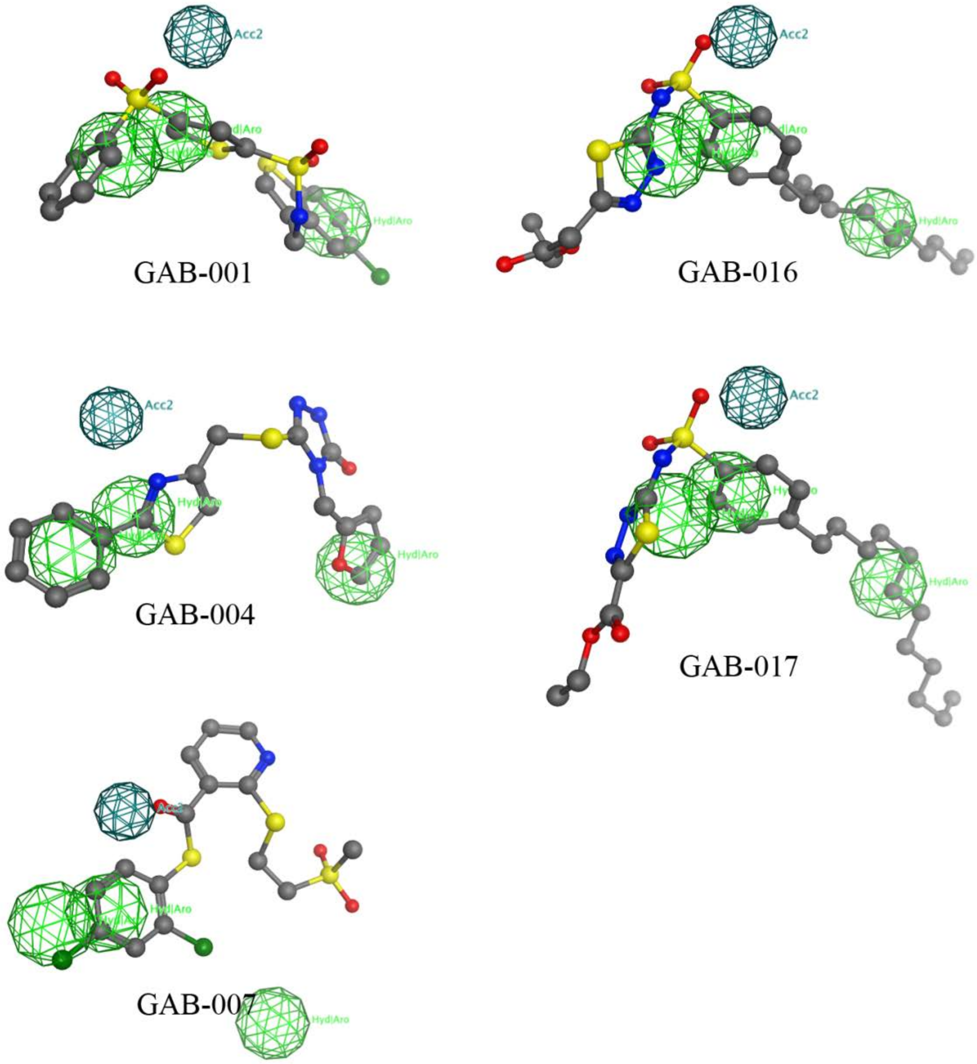

Supplement: S12 Fig — Pharmacophore of the inhibitors. Green: hydrophobic or aromatic region. Blue: the projection of H-bond acceptor. (PDF) [file pcbi.1004021.s012.pdf]
