# Supplementary material for: Novel Inhibitors Induce Large Conformational Changes of GAB1 Pleckstrin Homology Domain and Kill Breast Cancer Cells
Source: PLoS Comput Biol. 2015 Jan 8;11(1):e1004021. doi: 10.1371/journal.pcbi.1004021 (PMC4287437; doi:10.1371/journal.pcbi.1004021)
Supplement: S1 Table — Docking scores for 20 htis. The hits which are consistently active in three assays are labeled with bold IDs. (PDF) [file pcbi.1004021.s013.pdf]

Table S1. Docking scores for 20 hits. The hits which are consistently active in three assays are labeled with bold IDs.

Compound ID	Structure	ChemPLP
**GAB-001**	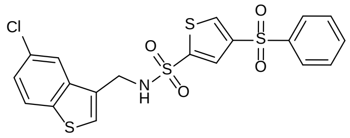	83.87
GAB-002	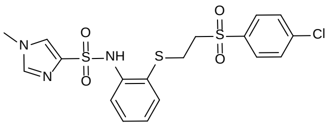	78.18
GAB-003	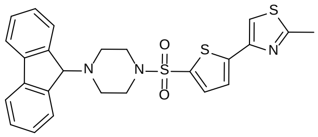	81.7
**GAB-004**	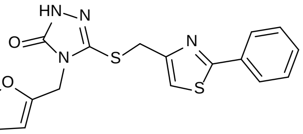	75.44
GAB-005	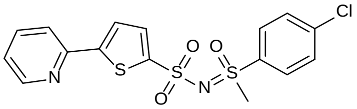	80.45
GAB-006	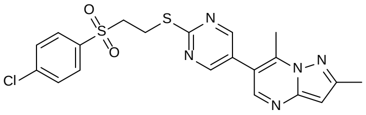	82.91
**GAB-007**	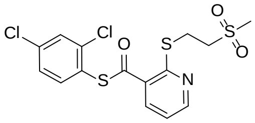	78.68
GAB-008	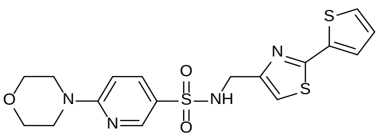	75.72
GAB-009	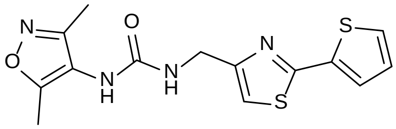	73.3

GAB-010	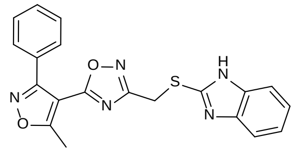	79.14
GAB-011	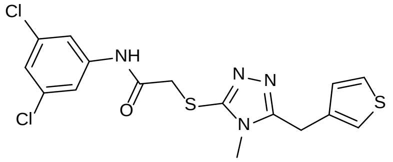	75.34
GAB-012	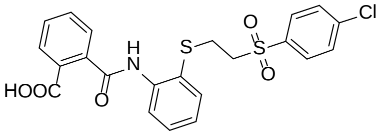	82.3
GAB-013	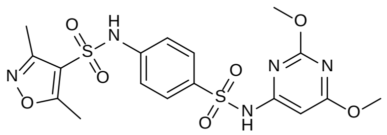	62.42
GAB-014	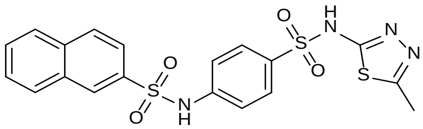	80.07
GAB-015	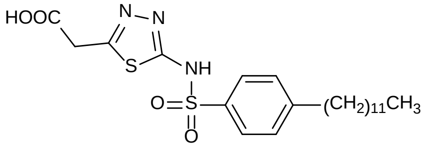	82.98
**GAB-016**	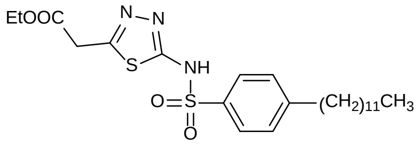	89.07
**GAB-017**	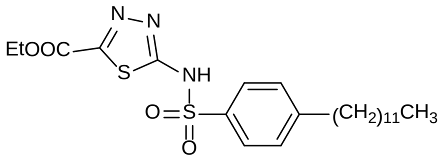	88.24
GAB-018	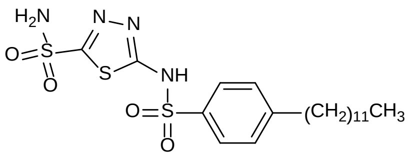	85.26
GAB-019	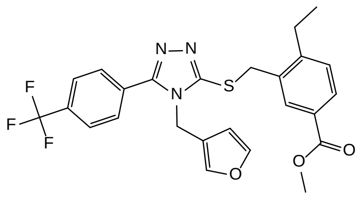	103.92

GAB-020	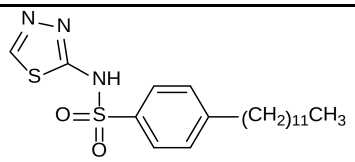	83.18
DPIEL	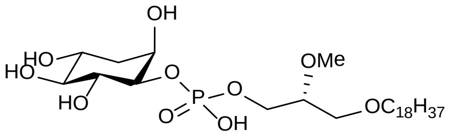	NA
IP4	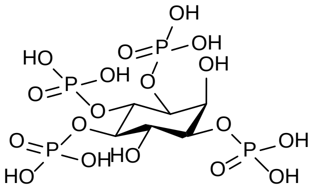	NA
