# Supplementary material for: Novel Inhibitors Induce Large Conformational Changes of GAB1 Pleckstrin Homology Domain and Kill Breast Cancer Cells
Source: PLoS Comput Biol. 2015 Jan 8;11(1):e1004021. doi: 10.1371/journal.pcbi.1004021 (PMC4287437; doi:10.1371/journal.pcbi.1004021)
Supplement: S2 Table — The crystal structures employed to generate PSSM. (PDF) [file pcbi.1004021.s014.pdf]

Table S2. The crystal structures employed to generate PSSM.

PDB ID	Protein name
3UZT	GRK2
1EAZ	TAPP1
1FAO	DAPP1
1W1D	PDPK1
1U27	ARNO
1FHW	GRP1
1UPR	Pepp1
3AJ4	Evectin-2
4FJH	Kindlin-2
2DYN	Dynamin-1
2DTC	RalGPS1
3PP2	ArhGAP27
2P0H	ArhGAP9
3NSU	SLM1
3CXB	SKIP
3HK0	Grb10
1QQG	IRS1
1UNQ	AKT1
1XD4	SOS homolog 1
1LB1	Dbs
3KZ1	PRG
1KI1	Intersectin
1FOE	Tiam1
2W2X	PLCG2
2VSZ	ELMO1
1MAI	PLCD1
3TW1	Rtt1066
2R2Y	Rpn13
3VOQ	Sin1
3U12	USP37
3PEG	Neurofibromin
4EMO	SHARPIN
4DIX	SCAB1
3RCP	Fapp1
