# Supplementary material for: Novel Inhibitors Induce Large Conformational Changes of GAB1 Pleckstrin Homology Domain and Kill Breast Cancer Cells
Source: PLoS Comput Biol. 2015 Jan 8;11(1):e1004021. doi: 10.1371/journal.pcbi.1004021 (PMC4287437; doi:10.1371/journal.pcbi.1004021)
Supplement: S3 Table — PSSM profile for PH domain (β1, β2, β3, β6, β7 and α1). (PDF) [file pcbi.1004021.s015.pdf]

Table S3. PSSM profile for PH domain (β1, β2, β3, β6, β7 and α1).

β1

	A	R	N	D	C	Q	E	G	H	I	L	K	M	F	P	S	T	W	Y	V
**-2**	-2	-2	-2	0	1	-2	0	-1	2	0	0	0	1	1	3	-2	-2	-3	1	1
**-1**	-1	1	-1	1	-3	1	1	-3	2	0	0	1	-1	-2	-2	0	1	-2	2	0
**1**	-1	0	-2	-2	-3	1	1	-3	3	0	0	3	2	0	-3	-1	-2	-3	-2	1
**2**	1	-1	-1	0	-3	1	2	3	2	-3	-3	1	0	0	-3	1	0	-4	-2	-2
**3**	-2	-3	-2	0	-4	-3	-1	4	1	0	-1	-3	-2	2	0	0	-1	-3	-2	0
**4**	-2	-1	1	-3	1	0	-2	-4	3	-2	1	0	1	1	0	-1	-1	4	3	0
**5**	0	-1	-1	-4	2	-3	-3	-1	1	2	2	-3	1	1	-4	-2	-2	-3	1	1
**6**	-1	2	-2	-1	-3	-1	-1	-1	4	-2	0	0	2	-2	1	1	0	4	0	0
**7**	-3	2	-3	-4	-4	-2	-2	-4	-3	1	0	4	1	-3	-4	-3	1	4	0	2
**8**	-1	3	-1	0	-3	1	-1	-1	-2	0	0	2	2	0	-1	-2	0	3	-2	0

β2

	A	R	N	D	C	Q	E	G	H	I	L	K	M	F	P	S	T	W	Y	V
**1**	-1	1	1	2	-4	-2	0	-3	2	-4	-4	-2	-3	-2	0	-1	1	9	2	-4
**2**	0	1	-2	-2	-4	0	-1	-1	-2	-3	-2	5	0	0	1	1	1	-4	-3	-3
**3**	-1	3	-1	1	-4	2	3	-3	1	-1	-3	2	-2	-1	1	-1	-1	-4	-2	-3
**5**	-2	3	-1	-3	0	-2	-2	1	3	-2	1	-1	-1	1	-3	0	-2	3	2	0
**6**	-1	0	-3	-4	-4	1	-3	-4	4	-3	0	-3	-2	3	-4	0	-1	8	4	-3
**7**	-1	-4	-4	-4	4	-3	-4	-2	-3	1	2	-4	0	4	-4	-3	-1	-2	1	2
**8**	-1	0	-2	-1	0	0	-1	-1	1	0	1	0	-1	3	-3	-2	0	-2	2	1
**9**	-3	0	-5	-5	-3	-4	-4	-5	-4	3	4	-3	0	2	-4	-4	-2	-3	-2	2

β3

	A	R	N	D	C	Q	E	G	H	I	L	K	M	F	P	S	T	W	Y	V
**1**	1	1	-1	-3	2	-1	-1	-1	3	0	0	0	-2	0	-3	1	1	-3	-2	0
**2**	-3	-1	-5	-6	-3	-4	-5	-6	-5	3	4	-4	2	2	-5	-4	-3	-4	-2	3
**3**	-2	-2	-3	-3	2	0	1	-2	2	2	1	-1	-1	0	-3	0	-1	3	3	1
**4**	-3	-4	-4	-4	5	1	0	-2	-3	0	1	-4	-2	3	-5	-3	-3	6	5	0
**5**	-2	-1	-3	0	4	-1	-1	-1	1	-3	-2	0	-2	3	-4	0	0	-2	5	-1
**+1**	-2	0	-2	2	-5	0	0	0	-2	-4	-4	4	-3	0	1	1	0	-3	3	-4

β6

	A	R	N	D	C	Q	E	G	H	I	L	K	M	F	P	S	T	W	Y	V
**1**	0	1	1	-2	3	1	-2	0	-2	1	0	1	2	-2	-3	-1	1	-3	-2	0
**2**	-2	-4	-5	-5	1	-4	-5	-5	1	0	3	-5	-1	7	-5	-4	-3	-2	2	0
**3**	0	-2	-2	-2	2	1	2	-3	2	0	0	0	-1	1	-3	0	1	-2	2	0
**4**	0	-4	-5	-5	-3	-4	-4	-5	-5	4	4	-4	0	-2	-4	-3	0	5	-2	1
**5**	-2	1	0	-2	-3	2	1	-3	3	1	0	1	-1	0	-3	-1	0	2	1	1

β7

	A	R	N	D	C	Q	E	G	H	I	L	K	M	F	P	S	T	W	Y	V
**1**	-2	2	2	-2	2	1	2	-3	3	-1	-3	2	0	-4	3	0	-2	-5	-3	0
**2**	-2	0	1	-2	1	1	2	0	2	-1	-1	-1	-2	0	1	0	1	-4	-2	1
**3**	-4	1	-5	-5	-4	-4	-4	-5	-3	1	2	-4	-2	4	-5	-4	0	4	5	0
**4**	-1	0	0	-1	-3	-1	1	-3	0	0	1	-1	-1	0	-3	0	1	3	2	1
**5**	-3	-5	-5	-5	-4	-5	-5	-5	-4	3	3	-5	-1	6	-5	-1	-3	-3	-1	0
**6**	0	0	-2	0	4	2	0	-3	3	-1	-2	0	2	-3	-3	1	-1	4	-2	-1
**+1**	3	-3	-2	0	4	-2	-2	-2	2	0	-2	-2	2	1	-3	0	1	-3	-2	0
**+2**	-2	-2	-1	4	-4	2	2	0	-2	-3	-1	0	-2	-4	3	0	-2	-4	-3	-1

α1

	A	R	N	D	C	Q	E	G	H	I	L	K	M	F	P	S	T	W	Y	V
**1**	-2	-2	2	2	-4	-2	-2	-3	3	-4	-4	2	-3	-4	2	3	3	-5	-3	-4
**2**	0	1	-1	0	-2	1	0	0	-1	0	-1	0	-1	-2	1	0	0	-2	1	0
**3**	1	-1	0	1	-3	1	3	-3	2	-3	-3	1	-3	0	0	0	0	-4	-2	-2
**4**	-3	-2	1	3	-5	2	5	-4	3	1	-4	-2	-3	-5	-3	1	0	-5	-4	-3
**5**	2	2	-3	-1	2	0	-2	-3	-2	0	1	1	2	-2	-3	-2	-2	-3	2	0
**6**	0	0	1	1	1	1	1	-2	2	0	-1	0	1	-1	-2	0	0	-3	-2	-1
**7**	1	0	0	0	-3	1	2	-1	2	-1	-1	1	-2	-3	-2	1	1	-4	-2	-2
**8**	-1	-6	-6	-7	-5	-5	-6	-6	2	1	-4	-6	-4	1	-6	-5	-5	11	-1	-4
**9**	-1	0	-2	-1	2	-2	-2	-4	2	2	0	0	4	1	-3	-2	0	-3	0	1
**10**	0	2	0	1	-3	2	0	-3	-2	-2	0	2	1	0	-3	-1	0	-3	-2	0
**11**	2	-2	0	-2	-2	-1	0	0	1	-1	0	1	-1	0	-2	1	0	-3	1	-1
**12**	0	-4	-5	-5	1	-4	-4	-5	-4	3	4	-4	0	1	-4	-3	-2	-3	-2	3
**13**	-1	4	2	1	0	2	0	-1	1	-1	-1	2	-2	-4	-3	-1	-2	-4	-3	-3
**14**	1	2	-1	1	-3	2	1	0	0	-1	-1	1	-2	-2	-2	0	0	2	0	-2
**15**	2	-2	-3	-3	1	-2	-3	-1	-3	2	1	0	-1	1	-3	0	-1	-3	-2	2
