# Supplementary material for: Novel Inhibitors Induce Large Conformational Changes of GAB1 Pleckstrin Homology Domain and Kill Breast Cancer Cells
Source: PLoS Comput Biol. 2015 Jan 8;11(1):e1004021. doi: 10.1371/journal.pcbi.1004021 (PMC4287437; doi:10.1371/journal.pcbi.1004021)
Supplement: S1 Method — Technical details for computational modeling. (PDF) [file pcbi.1004021.s016.pdf]

Method S1

PMF calculations

The explicit derivation of this PMF method has been described elsewhere [1,2]. It is a statistical mechanics methodology which calculates the binding free energy by introducing hypothetical intermediate states representing the association pathway of ligand from the unbound "bulk" regions to the ligand-binding "site". The average structure obtained from above 50 ns MD simulations was subject to energy minimization to remove structural defects. The resulted protein-ligand complex structure was employed as the reference frame to define the position and orientation restraints, as illustrated in **Fig. S9**. The position of the ligand with respect to its receptor protein is defined in a spherical coordinate system (r,θ,ϕ), whereas the orientation of the ligand is defined by a series of Euler angles (Θ,Φ,Ψ). r is the L1-P1 distance; θ is the L1-P1-P2 angle; ϕ is the L1-P1-P2-P3 dihedral angle; Θ is the P1-L1-L2 angle; Φ is the P1-L1-L2-L3 dihedral angle; Ψ is the P2-P1-L1-L2 dihedral angle. The harmonic potentials were applied to restrain the orientational and axial degrees of freedom with $u_o(\Theta,\Phi,\Psi)$ and $u_a(\theta,\phi)$, where $u_o(\Theta,\Phi,\Psi)=\frac{1}{2}k_o[(\Theta-\Theta_{ref})^2+(\Phi-\Phi_{ref})^2+(\Psi-\Psi_{ref})^2]$ and $u_a(\theta,\phi)=\frac{1}{2}k_a[(\theta-\theta_{ref})^2+(\phi-\phi_{ref})^2]$, respectively. The choice of atoms to define L1, L2, L3, P1, P2, P3 can be arbitrary, as validated before [3]. However, we try to standardize the definition of these points as following: L1 is the ligand center of mass; L2 and L3 are two terminal moieties relative to L1; P1 is the center of mass of the conserved L45, F85 and W107 (GAB1) or L44, I95 and W106 (IRS1); P2 is the center of mass of the proline residues in $\beta_{1,2}$ loop; P3 is the center of mass of $\beta_{6,7}$ loop.

The term G_c^{bulk}, corresponding to the conformational restraints on the ligand free in solution, was calculated by Eq. **1**, in which $w_c^{bulk}(\xi)$ is the PMF as a function of ξ (the mass-weighted RMSD with respect of the reference ligand conformation). $w_c^{bulk}(\xi)$ was simulated by umbrella sampling in the presence of a conformational restraint with harmonic potential $u_c=\frac{1}{2}k_c(\xi[ligand;ligand_{ref}])^2$ and without the orientational and axial restraints, using the force constant k_c = 2 kcal/mol·Å^2. The umbrella sampling simulation for $w_c^{bulk}(\xi)$ were separated by 0.2 Å, and for each window, we performed 1 ns production simulation followed by 0.2 ns equilibration. For the ligands with dodecyl moiety (GAB-016 and GAB-017), the maximum RMSD was 8 Å, corresponding to a total of 40 umbrella sampling windows. Otherwise, the maximum RMSD was 6 Å (totally 30 windows). The PMF in the bulk was calculated with the weighted histogram analysis method (WHAM) [4].

Similarly, G_c^{site} (the conformational restraints on the ligand in the binding site) was calculated by Eq. **2**. The corresponding PMF $w_c^{site}(\xi)$ was computed with the same parameters and methodology employed when computing $w_c^{bulk}(\xi)$, except that the umbrella sampling simulations were done in the presence of protein.

$$e^{-\beta G_c^{bulk}} = \frac{\int d\xi e^{-\beta[w_c^{bulk}(\xi)+u_c(\xi)]}}{\int d\xi e^{-\beta w_c^{bulk}(\xi)}}, \qquad (1)$$

$$e^{-\beta G_c^{site}} = \frac{\int d\xi e^{-\beta[w_c^{site}(\xi)+u_c(\xi)]}}{\int d\xi e^{-\beta w_c^{site}(\xi)}}, \qquad (2)$$

For umbrella sampling simulations along the axis r, the window configurations were generated with a biasing radial potential $u(r) = \frac{1}{2}k_r(r-r')^2$, in which the force constant k_r = 2 kcal/mol·Å^2. The windows were spaced by 0.5 Å, and the maximum L1-P1 distance (r*) was 40 Å. Of note, the r* is an arbitrary value, but it does not affect the final binding free energy value according to previous assessment [3]. The umbrella sampling simulations were done in the presence of the positional and orientational restraints. To accommodate the possible conformational changes during ligand separation, very soft harmonic potentials were applied on orientational and axial restraints, with $k_a = k_o$ = 0.2 kcal/mol·rad^2. We performed 0.5 ns production simulation followed by 0.2 ns equilibration for each window. The resulted PMF along r axis, $w(r)$, was used to calculate the separation PMF (I*) by integration of the Boltzmann constant (Eq. **5**).

Other terms, such as S* and G_o^{bulk}, were calculated from Eq. **3** and Eq. **4** by direct numerical integrations. Different from the original work, the contribution of free energy costs of orientational restriction (G_o^{site}) and axial restriction (G_a^{site}) in the binding site were ignored, as a very soft force constant (0.2 kcal/mol·rad^2) was used. Still, the sum of G_o^{site} and G_a^{site} was estimated at an order of 0.01 kcal/mol using Eq. **6**, assuming the PMF for any angular or torsional restraints is similar with that for the original work [3]. X in Eq. **6** represents any angular degree of freedom, including $\theta, \phi, \Theta, \Phi, \Psi$.

$$S^* = (r^*)^2 \int_0^{\pi} \sin(\theta) d\theta \int_0^{2\pi} d\phi e^{-\beta u_a(\theta,\phi)}, \qquad (3)$$

$$e^{-\beta G_o^{bulk}} = \frac{1}{8\pi^2} \int_0^{\pi} \sin(\Theta) d\Theta \int_0^{2\pi} d\Phi \int_0^{2\pi} d\Psi e^{-\beta u_o(\Theta,\Phi,\Psi)}, \qquad (4)$$

$$I^* = \int_{site} dr e^{-\beta[w(r)-w(r^*)]}, \qquad (5)$$

$$e^{-\beta G_X^{site}} = \frac{\int dX e^{-\beta[w_X^{site}(X)+\frac{1}{2}k_o(X-X_{ref})^2]}}{\int dX e^{-\beta w_X^{site}(X)}} = 0.9992 \Rightarrow G_X^{site} \approx 0.0005 kcal/mol, \qquad (6)$$

The final binding free energy ΔG_{bind} was calculated using Eq. 7, where $C°$ is the standard state concentration of 1 mol/L (≡ 1/1,661 Å^3)

$$\Delta G_{bind} = -\frac{1}{\beta}\ln(S * I * C^{\circ}) + G_c^{bulk} + G_o^{bulk} \overbrace{-G_o^{site} - G_a^{site}}^{<0.01 kcal/mol} - G_c^{site} \approx -\frac{1}{\beta}\ln(S * I * C^{\circ}) + G_c^{bulk} + G_o^{bulk} - G_c^{site} \quad (7)$$

Reference List

1. Woo HJ, Roux B (2005) Calculation of absolute protein-ligand binding free energy from computer simulations. Proceedings of the National Academy of Sciences of the United States of America 102: 6825-6830.

2. Lau AY, Roux B (2011) The hidden energetics of ligand binding and activation in a glutamate receptor. Nature Structural & Molecular Biology 18: 283-U62.

3. Gumbart JC, Roux Bt, Chipot C (2012) Standard Binding Free Energies from Computer Simulations: What Is the Best Strategy? J Chem Theory Comput . doi: 10.1021/ct3008099.

4. Kumar S, Rosenberg JM, Bouzida D, Swendsen RH, Kollman PA (1992) THE weighted histogram analysis method for free-energy calculations on biomolecules. I. The method. J Comput Chem 13: 1011-1021. 10.1002/jcc.540130812.
